# The provenance of the raw material and the manufacturing technology of copper artefacts from the Copper Age hoard from Magyaregres, Hungary

**DOI:** 10.1371/journal.pone.0278116

**Published:** 2022-11-23

**Authors:** Zsuzsanna Siklósi, Eszter Horváth, Igor Maria Villa, Stefano Nisi, Viktória Mozgai, Bernadett Bajnóczi, Péter Csippán, Péter Hornok, Péter Kiss

**Affiliations:** 1 Institute of Archaeological Sciences, Eötvös Loránd University, Budapest, Hungary; 2 Institute of Geology, University of Bern, Bern, Switzerland; 3 Centro Universitario Datazioni e Archeometria, Università di Milano Bicocca, Milano, Italy; 4 Laboratori Nazionali del Gran Sasso, Instituto Nazionale di Fisica Nucleare, L’Aquila, Italy; 5 Institute for Geological and Geochemical Research, Research Centre for Astronomy and Earth Sciences, Eötvös Loránd Research Network (ELKH), Budapest, Hungary; 6 CSFK, MTA Centre of Excellence, Budapest, Hungary; 7 Vas County Government Office Department of Construction and Heritage Protection, Szombathely, Hungary; 8 Savaria Museum, Szombathely, Hungary; New York State Museum, UNITED STATES

## Abstract

In 2016, a Stollhof-type copper hoard was found during an excavation in Magyaregres, Hungary. It was placed in a cooking pot, and deposited upside down within the boundaries of an Early Copper Age settlement. Similar hoards dating to the end of the 5^th^ millennium BCE are well-known from Central Europe, however, this hoard represents the only one so far with thoroughly documented finding circumstances. The hoard contained 681 pieces of copper, 264 pieces of stone and a single *Spondylus* bead, along with 19 pieces of small tubular spiral copper coils, three spiral copper bracelets, and two large, spectacle spiral copper pendants. Until now, information on the provenance of raw materials and how such copper artefacts were manufactured has not been available. The artefacts were studied under optical microscopes to reveal the manufacturing process. Trace elemental composition (HR-ICP-MS) and lead isotope ratios (MC-ICP-MS) were measured to explore the provenance of raw materials. The ornaments were rolled or folded and coiled from thin sheets of copper using fahlore copper probably originating from the Northwestern Carpathians. A complex archaeological approach was employed to reveal the provenance, distribution and the social roles the ornaments could have played in the life of a Copper Age community. Evidence for local metallurgy was lacking in contemporaneous Transdanubian sites, therefore it is likely that the items of the hoard were manufactured closer to the raw material source, prior to being transported to Transdanubia as finished products. The method of deposition implies that such items were associated with special social contexts, represented exceptional values, and the context of deposition was also highly prescribed. The Magyaregres hoard serves as the first firm piece of evidence for the existence of a typologically independent Central European metallurgical circle which exploited the raw material sources located within its distribution.

## Introduction

The chronological terminologies concerning Southeast Europe and the Carpathian Basin refer to the second half of the 5^th^ millennium cal BCE as the Copper Age; a time of major technological, economic and social change [1–4]. In the definition of the Copper Age as an independent chronological period, the appearance of heavy copper objects made of pure copper was particularly significant [5,6]. Academic research of the 19^th^ century corresponded the beginning of this period with the appearance of large volumes of copper artefacts in the archaeological record. However, research today takes the stages of technological advances along with social changes into consideration–the emergence of complex societies, the increase of social inequalities and–economic changes in which metallurgy could have played a crucial social and cultural role [2–4,7–13].

The origins of Southeast European metallurgy stretch as far back as the Neolithic [4,14–16]. Emerging out of these traditions, the significance of the Balkan metallurgical circle grew substantially by the Early Copper Age [15,17–23] to the extent that its products distributed far and wide, to the northern regions of modern-day Germany and Denmark [24,25], and according to some, even to the coastal areas of the Atlantic [26]. The distribution of copper prestige goods outline a long-distance social interaction network between the regions of Southeast and Central Europe [24,26,27], which could have also facilitated the transmission of technological knowledge [28,29].

As a result of this process, towards the end of the 5^th^ millennium BCE in eastern Central Europe and the western Carpathian Basin the so-called Jordanów/Jordansmühl-Ludanice-Balaton-Lasinja culture complex came to existence [21]–characterised by a new metallurgical tradition producing distinct assemblages (consisting of spectacle spiral pendants, large spiral bracelets, gold, silver or copper discs with multiple bosses, tubular spiral coils etc.), typologically clearly distinguishable from the products of the Balkan metallurgical circle [21,30,31]. The studies exploring the chemical composition of these artefacts, along with a few lead isotope analyses (see for example the Hornstaad-Hörnle copper disc, Handlová ingot) [32–37], compared with the lead isotope ratios measured on copper ore sources in the Northwest Carpathians point toward the exploitation of local raw material sources in the territory of present-day Slovakia [34,38–40]. However, at the moment, the number of lead isotope analyses carried out on these types of ornaments is very small, whereas, the available lead isotope range measured on Slovakian copper ores is too broad to draw a meaningful comparison. In order to exclude certain raw material sources, lead isotope measurements were needed to be combined with chemical compositional analyses and contrasted with the archaeological record.

One of the first hoards representing this new metallurgical tradition from Central Europe was the accidentally discovered Stollhof hoard from Austria, consisting of copper and gold ornaments [41,42]. This hoard played a significant role later in the chronological definition of the Copper Age in the Carpathian Basin. Since then, a number of similar hoards came to light, the majority of these as stray finds, while the finding circumstances of others, unearthed largely during the course of the 19^th^ century, remain unknown (e.g. Csáford, Štramberk-Kotouč, Malé Leváre) [36,43–46]. The hoards of Hlinsko and Vanovice, although assigned to a slightly younger chronological horizon, represent two exceptions in this regard, deposited in ceramic vessels at hilltop settlements [31,47].

The Magyaregres hoard is the first one to be brought to light from a well-documented archaeological context from an Early Copper Age settlement in Southern Transdanubia, lending it outstanding significance. This site located far from potential raw material sources, therefore the overall aim of the project was to investigate the provenance of raw materials and the *chaîne opératoire* implemented during the manufacturing process. Our project was established to investigate where the raw material of copper artefacts derived from, how they were produced and whether the artefacts were made locally or reached the regions of Southern Transdanubia via long-distance social networks. Moreover, a detailed examination of the archaeological context was performed to reveal their social role. To achieve this, lead isotope analyses were combined with chemical compositional examinations for provenancing the raw materials. Macrostructure analysis was conducted on the artefacts in order to reveal the manufacturing techniques used in their production. Finally, accelerator mass spectrometry [AMS] radiocarbon measurements, followed by the archaeological contextualisation of the assemblage were carried out to interpret the scientific results. The outcomes of these investigations help to better understand how Copper Age metallurgy operated in Central Europe, furthermore, whether an independent Central European metallurgical circle exploiting its own raw material sources existed, and how the spread of technological knowledge can be reconstructed via long-distance networks of interactions.

### The early copper age in transdanubia and the Copper Age settlement of Magyaregres-Varga-Bonyi-ároktól keletre, Hungary

By around 4300 cal BCE the characteristic lifestyles that gave rise to Neolithic tell settlements came to an end in the Balkans and in the Carpathian Basin. After the cessation of tells along with large, single-layered settlements on the Great Hungarian Plain, a dense network of small-scale, independent hamlets was established. The location of these settlements shifted frequently [48,49]. The changes that took place at this time in Transdanubia correspond well with the transformation of settlement structure that occurred in the broader region of the Carpathian Basin. Although the final phase of the large, Late Neolithic Lengyel settlements and the Early Copper Age Lengyel III sites are still not dated precisely enough, the use of the latter almost certainly ceased by 4300 cal BCE [50,51]. Hungarian prehistoric research considered the subsequent period of the Balaton-Lasinja culture as Middle Copper Age, however, due to the increased number and availability of AMS radiocarbon dates and their Bayesian modelling, it has become clear that the first Balaton-Lasinja sites occur around 4350/4300 cal BCE–as opposed to 4000 cal BCE as it was previously thought–which corresponds well with the socio-economic changes taking place on the Great Hungarian Plain. Therefore, a change in terminology was suggested, classifying the Balaton-Lasinja sites as Early Copper Age [52–54].

At the same time, around 4350/4300 cal BCE, a dense network of small-sized hamlets was established by the Balaton-Lasinja and the Ludanice culture. However, whether these settlements were contemporaneous or emerged subsequently of each other remains unclear. The archaeological assemblages unearthed at these sites indicate the increased significance of animal husbandry and an overall more mobile lifestyle than in the previous period [21,55–57]. During this period in Transdanubia including the closer region of Budapest, settlements were founded in previously unoccupied territories, including hilltops, caves and small islands surrounded by marshland [12,21,52,57–62]. The ceramic styles and the construction of buildings in the northern regions of Transdanubia suggest close connections with the Ludanice culture distributing north of present-day Hungary [59,61,63], while ceramic styles occurring in the southern regions of Transdanubia (south of Lake Balaton) formed part of what is referred to as the Balaton-Lasinja culture in the academic literature [52,56,57,64]. However, there is no clear boundary between the two cultures [59,63] the differences are reflected mainly by ceramic styles.

**Fig 1:**
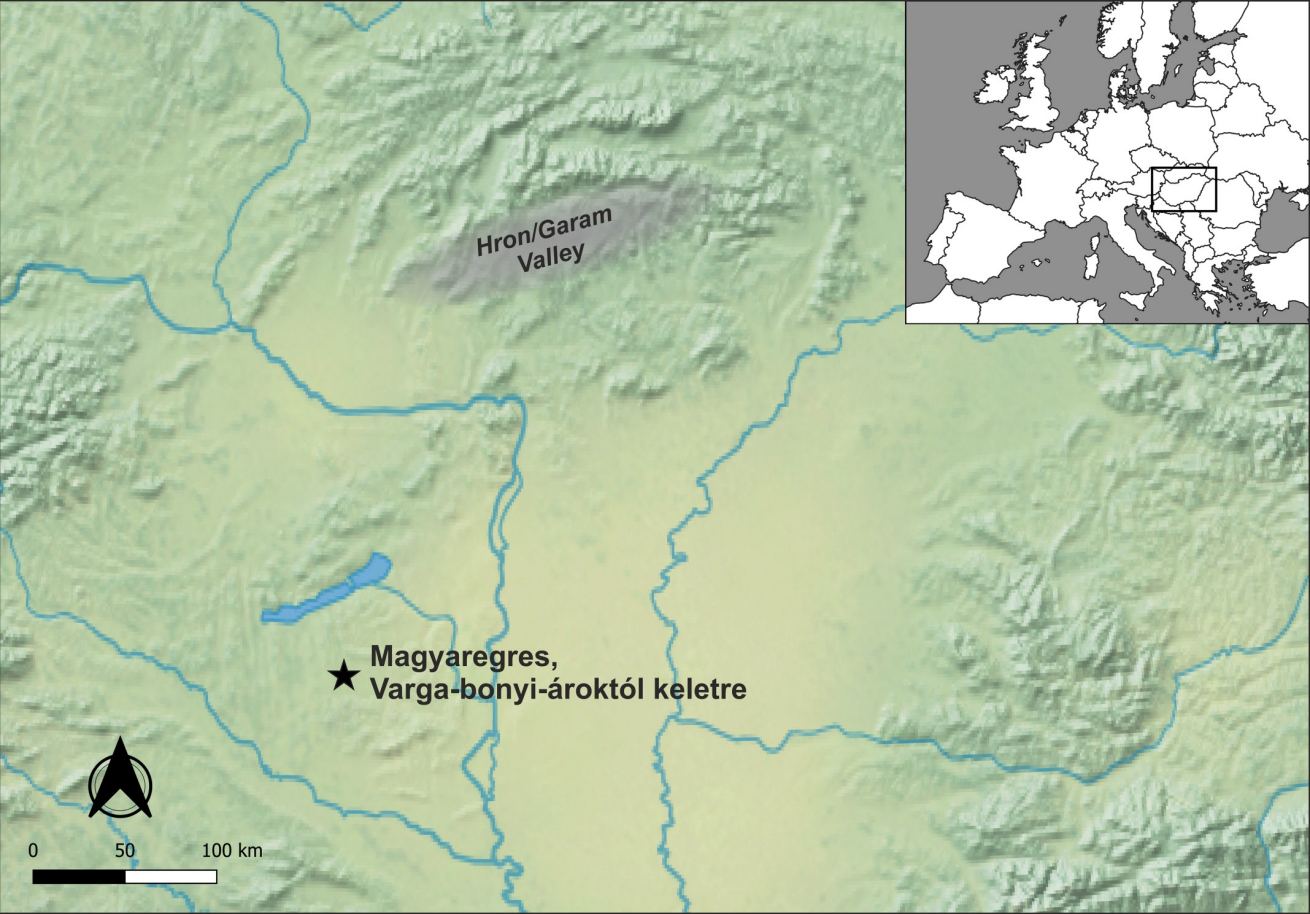
Map showing the location of the site and the area of supply for copper raw materials. The map is created using “Natural Earth. Free vector and raster map data” available at https://www.naturalearthdata.com/downloads/10m-raster-data/.

A similar, small-sized hamlet was unearthed at the site of Magyaregres-Varga-Bonyi ároktól keletre (46°27’19,6’’N, 17°46’42,7’’E) in 2016–2017, in southern Transdanubia prior to the construction of a motorway (Fig 1). The site is located on the western slopes of a hill between the Varga-Bonyi Valley and the Deseda Stream. On the hilltop, 155–163 meters above sea level, the Copper Age settlement associated with the Balaton-Lasinja culture was brought to light. Altogether eight, rectangular buildings constructed with foundation trenches were documented (7.9–9.7x11.7–13m) oriented in NW–SE direction. The construction and layout of the buildings were similar but not identical, two of the buildings were surrounded by a circular ditch, and one by a rectangular ditch. Two of the buildings had further structures attached to the main building. The buildings were constructed in close proximity to each other, while pits of various functions clustered in the NE and SW sectors of the excavation. The overall layout indicates a distinction between different functional areas within the hamlet [65]. The layout and the building structures show similarities with the contemporary settlement structures of Transdanubia, Lower Austria, Slovakia, Moravia and Croatia [52,58–60,62,66–69].

#### The copper age hoard

**Fig 2:**
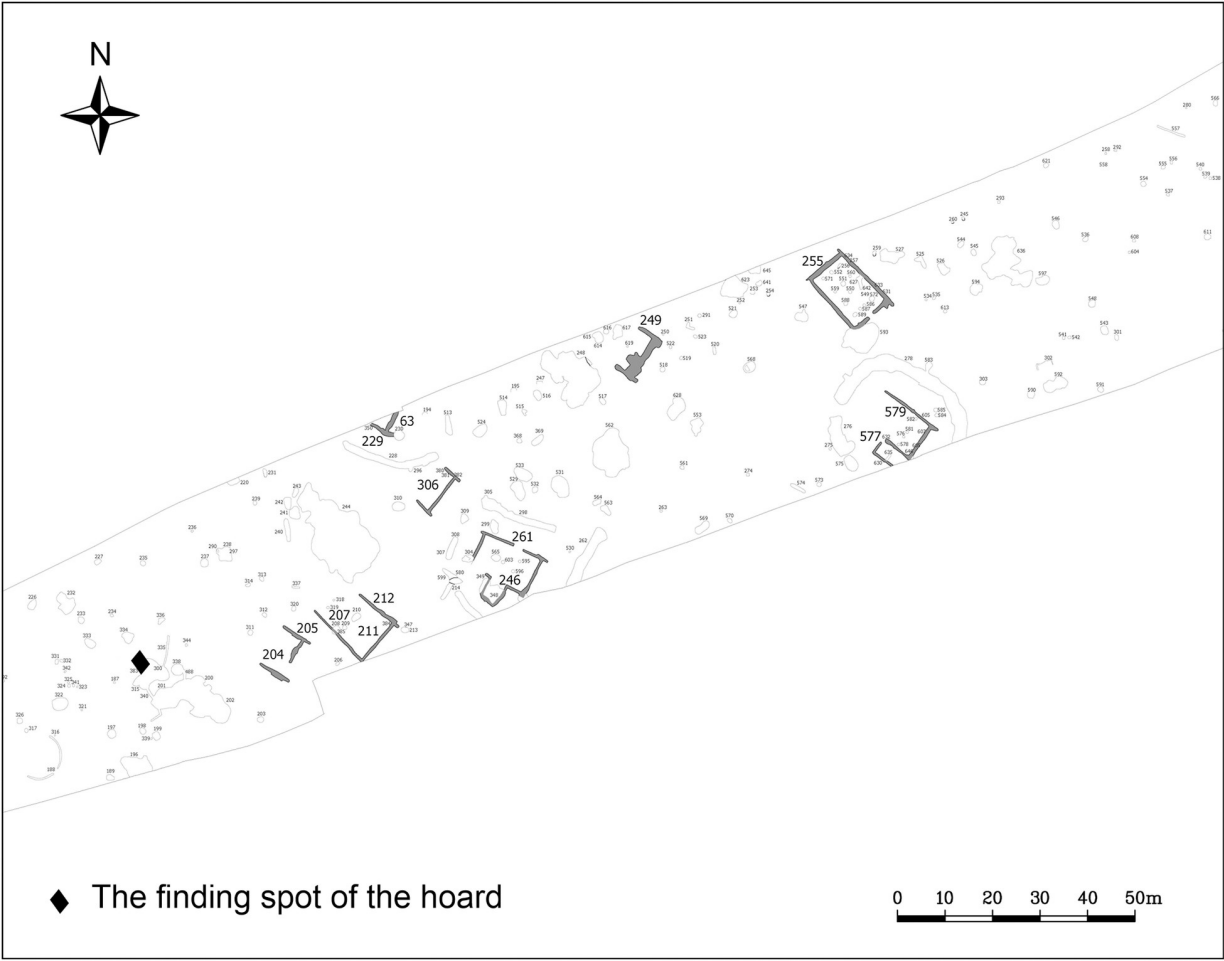
Location of the hoard at the site. Drawing: Archeoland Kft.

**Fig 3:**
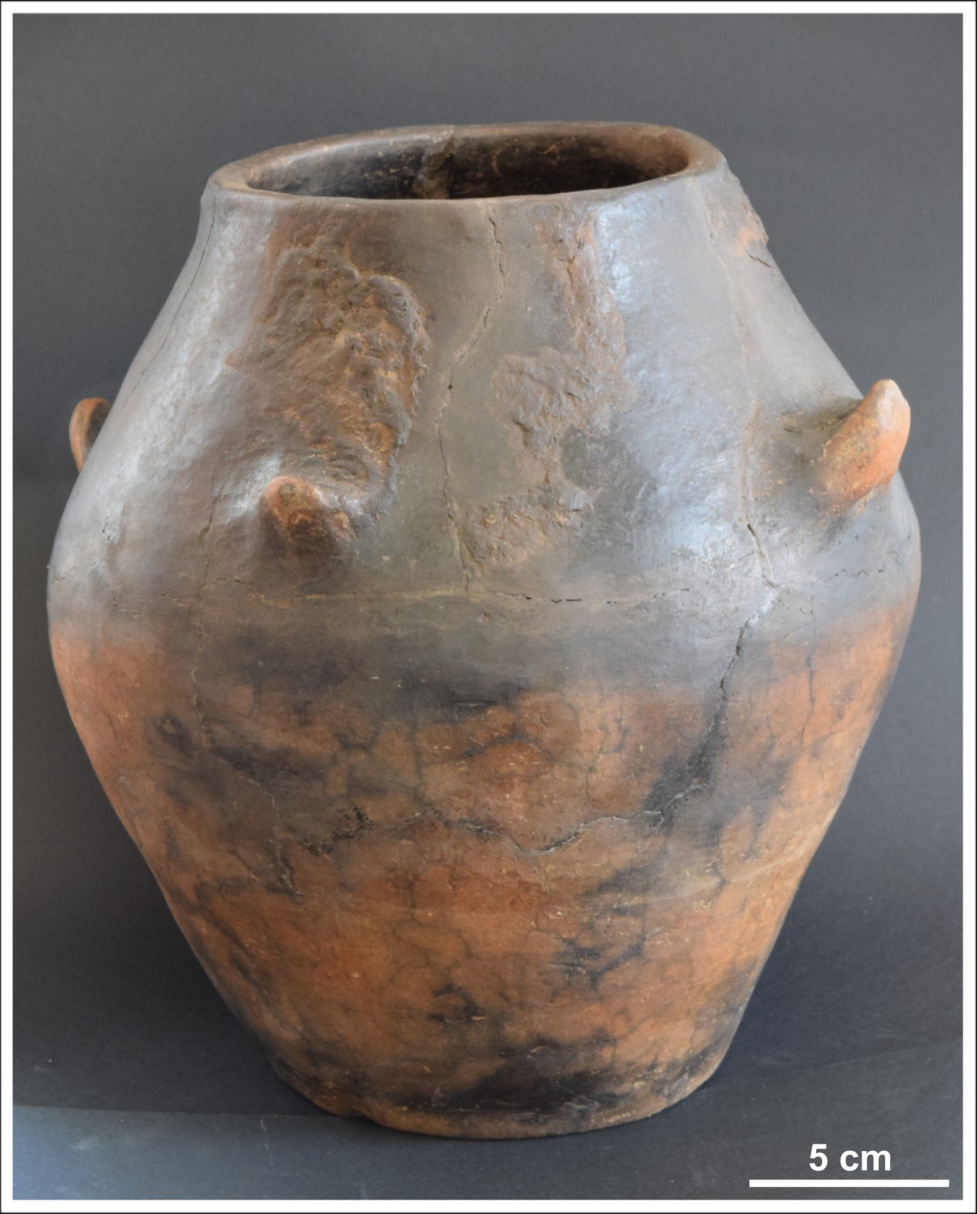
The ceramic vessel containing the hoard. Photo: Péter Kiss.

**Fig 4:**
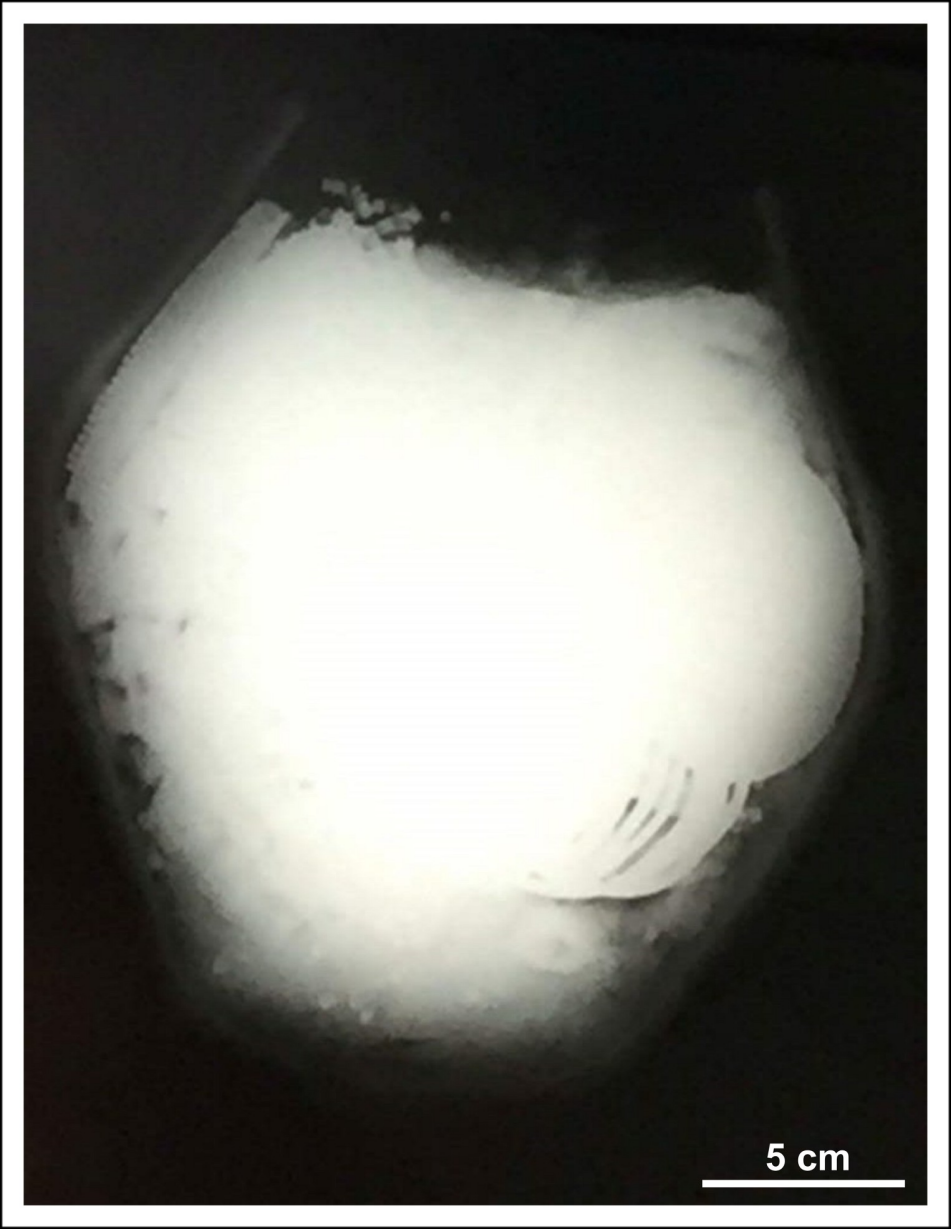
X-Ray image of the hoard.

**Fig 5:**
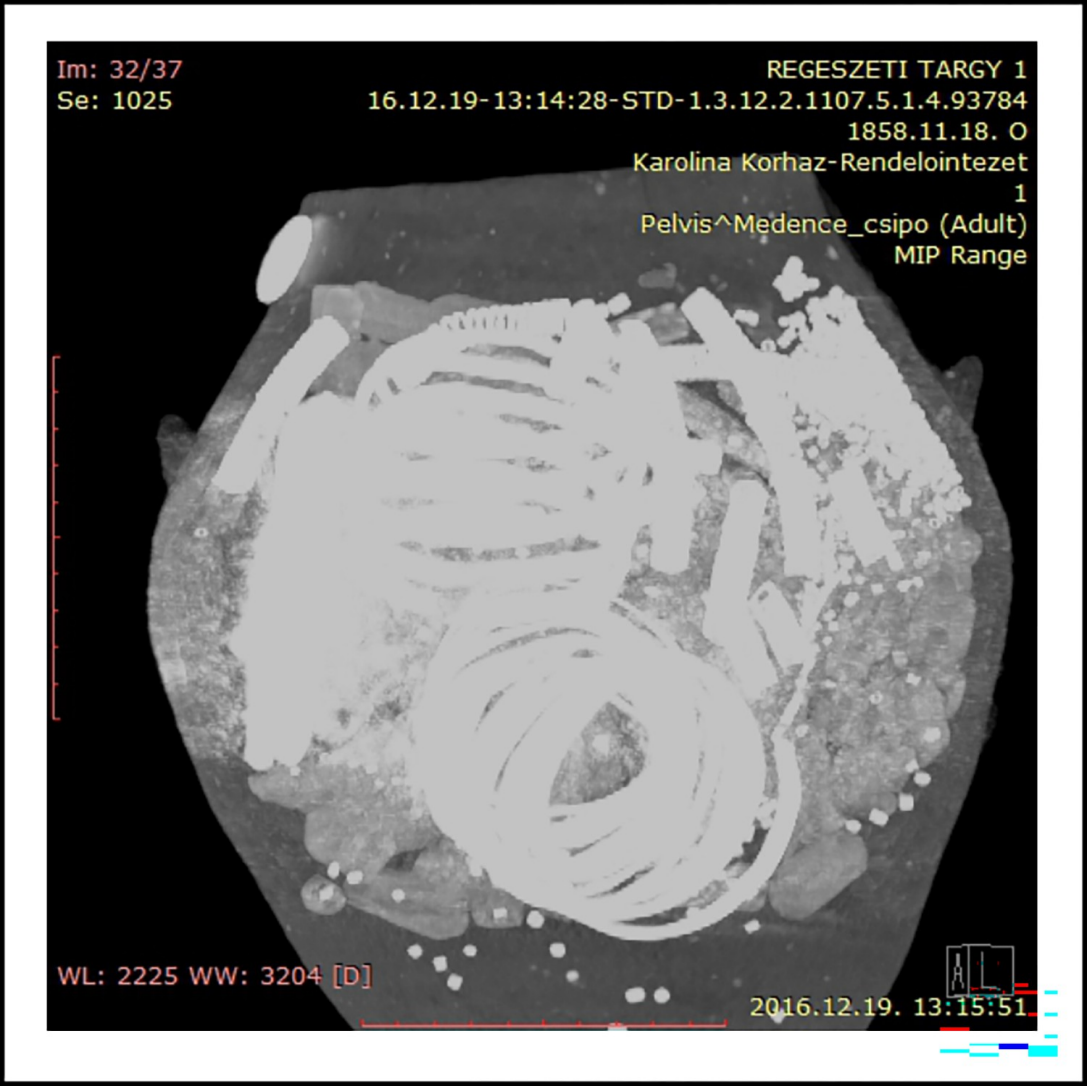
CT scan of the hoard.

On the western side of the Copper Age settlement an intact, biconical cooking pot was found deposited upside down (Figs 2 and 3; S1 File) in a refuse pit (stratigraphic unit no. 383). The vessel felt unusually heavy (11.4 kg) upon excavation, therefore an X-Ray (Fig 4) and computed tomography [CT] image (Fig 5) was arranged to be taken before it was emptied of its contents. The X-Ray image had undoubtedly shown a dense substance inside the pot. The CT scan revealed more details of the vessel’s contents making the careful retrieval of the artefacts possible. Furthermore, the CT image affirmed the presence of several larger and smaller spiral-like coils and a great number of small, cylindrical artefacts. The CT image had also shown–as it was later verified by the extraction of the pot’s contents–that the uppermost and lowermost segments of the vessel were empty, suggesting that the assemblage was wrapped in some kind of organic material. It is likely that the vessel’s orifice was also covered by organic material in order to prevent the objects from falling out after it was deposited upside down [65]. The order in which the items were placed in the vessel must have been carefully considered (Fig 5): the two spectacle spiral pendants were laid on top of each other while the small cylindrical beads were probably strung on a piece of string [65].

The vessel contained 265 pieces of beads (264 pieces of limestone, 1 piece of *Spondylus*), 681 pieces of small copper beads, 19 pieces of tubular spiral copper coils, three spiral copper bracelets (two of which were part of a single ornament) and two large spectacle spiral copper pendants (S2 File).

**Fig 6:**
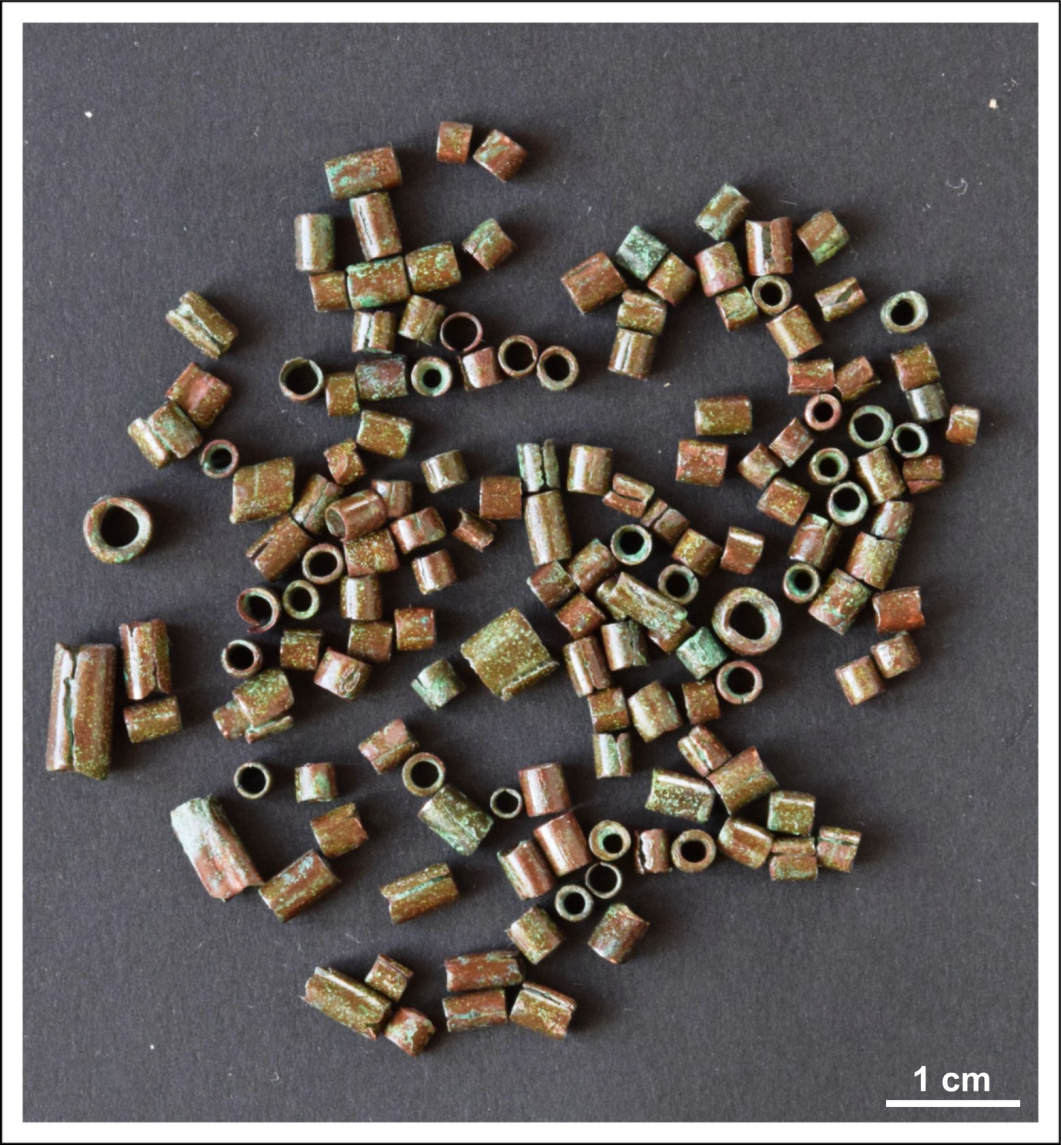
A selection of copper beads. Photo: Péter Kiss.

Some of the small cylindrical copper beads were slightly deformed, their lengths ranges between 2 and 6 mm, their diameters measure around 2.5–4 mm and their wall thickness varies between 0.05 and 0.5 mm. The wall thickness can vary even within the length of a single bead (Fig 6).

**Fig 7:**
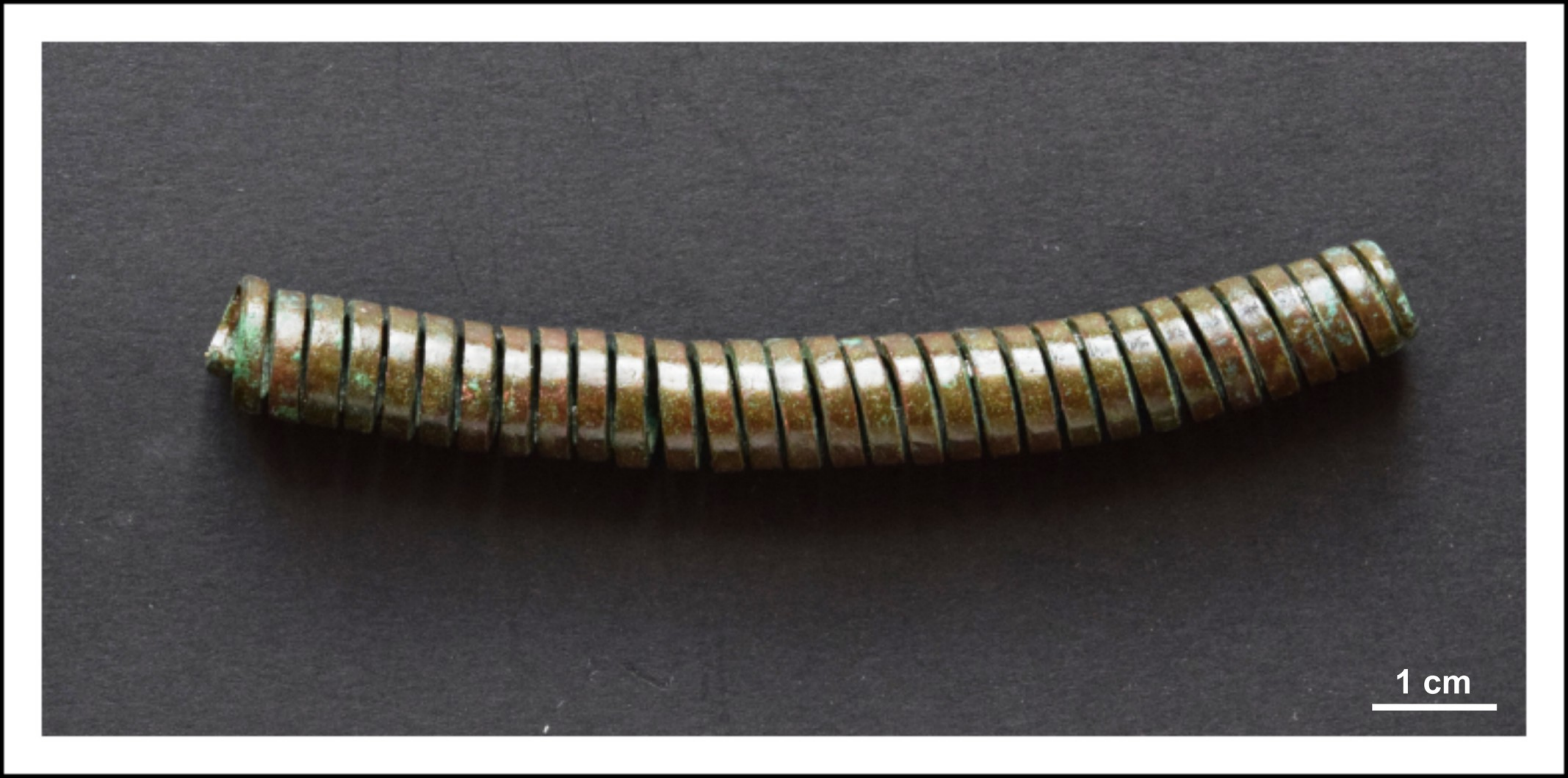
An example of tubular spiral coils. Photo: Péter Kiss.

The tubular spiral coils are very uniform both in their appearance and size–apart from their lengths. The length of the coils ranges between 16 and 95 mm, their outer diameter is around 9–10 mm, while the inner diameter is 5–6 mm. Their wall thickness varies between 0.75 and 1 mm (Fig 7).

**Fig 8:**
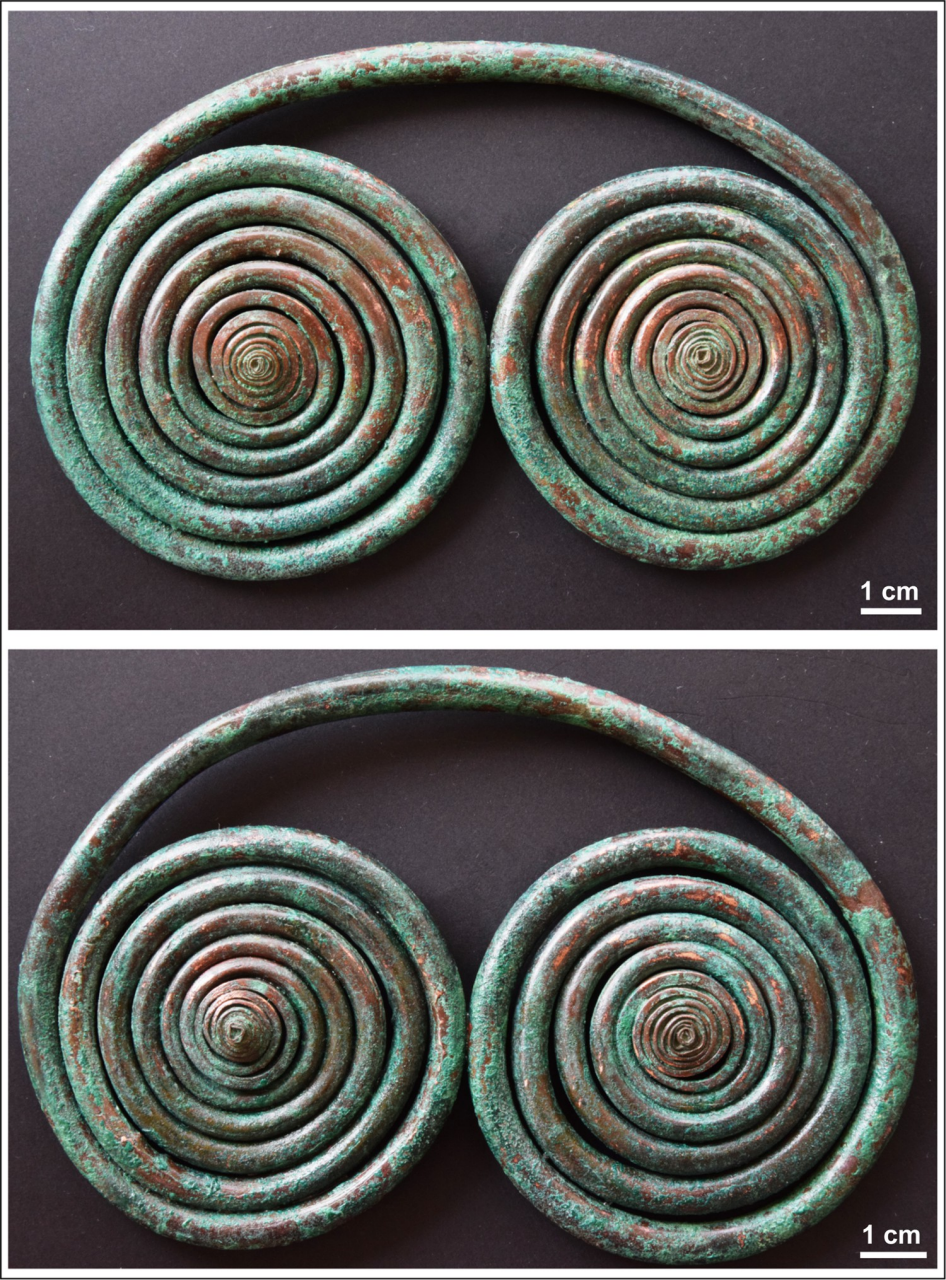
Spectacle spiral pendants. Photo: Péter Kiss.

The spectacle spiral pendants (as the name suggests) were created by rolling then coiling both ends of a piece of copper strip into a (double) disc-shaped ornament connected by an arching loop. The centres of both double spiral elements protrude slightly. The diameter of the coils increases from the centre towards the outer perimeters of the disc. The cross section of the coils is rectangular in the centre of the ornament, turning square before becoming completely rounded in the outer sections (Fig 8).

**Fig 9:**
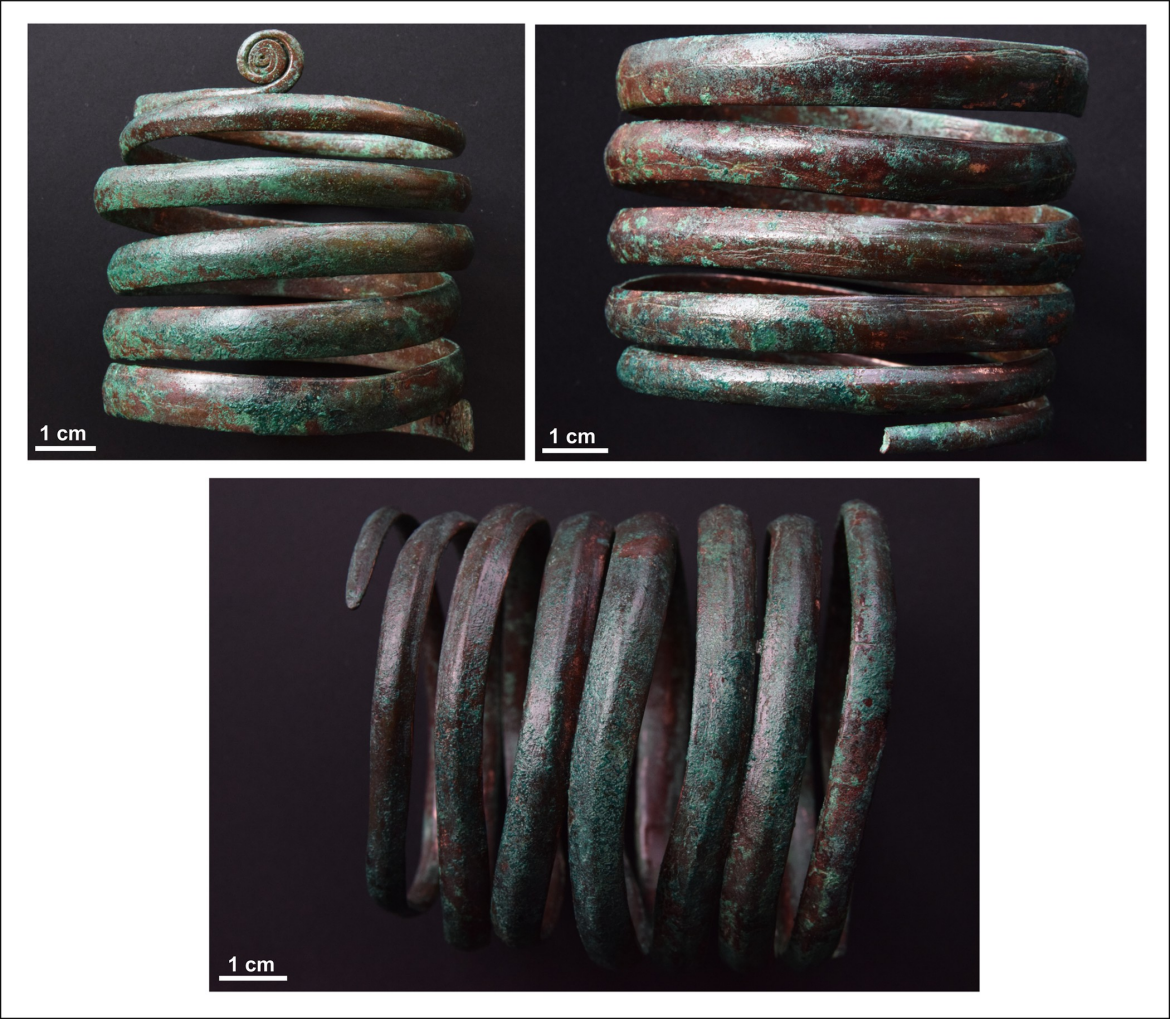
Spiral bracelets. Photo: Péter Kiss.

The hoard contained three individual spiral bracelets, two of which originally belonged together and formed a single ornament (Fig 9). The spiral bracelets were relatively similar both in their size and their appearance. In the case of the intact ornament, the ends of the copper coils were tapered and finished off in a small point. One of the ends of the other ornament (the one cut in half) were coiled into small disc. The diameter of the bracelets is very similar, the width of the individual coils decreases from the middle towards the two ends of the bracelet. In terms of the length, the bracelet cut in half was longer than the other ornament. The copper coil is semi-circular or lenticular in cross section in some places. The cross-section of the small, coiled disc-like finial starts out as being circular, and gradually becomes square before turning into oblong in the centre. In this regard, the execution of these ornaments is very similar to the coils of spectacle spiral pendants, apart from the disc-like finial, which in the case of the bracelets is flat, not protruding.

The hoard is held in the collection of the Rippl-Rónai Museum of Kaposvár, Hungary.

## Materials and methods

### X-Ray and computed tomography imaging

X-Ray images and CT scans taken of contents of the ceramic vessel prior to extraction at the Karolina Hospital in Mosonmagyaróvár aided the careful removal of the ornaments substantially. The X-Ray images were conducted on a digital ‛Top-X’ machine used for regular chest scans. The images arranged in 200x200 mm frames showed spiral-like objects and a dense substance (metal or stone) in the middle section of the cooking pot. Subsequent CT scans were carried out by a 16-slice Siemens Somatom Emotion CT imager. The vessel was scanned in both right way up and on its side with the following parameters: 130 kV, 23–247 mA, total mAs: 603, slice thickness: 1.0–3.0 mm. The images were analysed by using RadiAnt DICOM Viewer software.

### AMS dating and bayesian modelling

In order to date the archaeological site, samples for AMS radiocarbon dating were taken from settlement structures and animal bones. During the sampling process articulating and refitting animal bones were chosen for analysis, as it was more likely these dated the contexts in question more precisely than random pieces of debris (Table 1). Since the refuse pits of str. unit 383. and 340., and the hoard itself did not contain archaeological materials suitable for radiocarbon dating, we aimed to date the settlement as a whole. The AMS measurements were carried out by the Poznan Radiocarbon Laboratory, Poland. They were calibrated with the OxCal 4.4 software using the IntCal20 calibration curve [70,71]. The results were rounded up to 5 years and referenced with one sigma uncertainty. The radiocarbon dates were refined by Bayesian modelling during the calibration.

**Table 1:** The material and archaeological context of radiocarbon data.

Lab. no.	BP	δ^13^C*	Stratigraphic unit	Sample material
**Poz-120013**	5050±40	-20.4±0.3	S200-202	pig *maxilla* fragment, probably fits together with another *maxilla* fragment (from the same context)
**Poz-120014**	5290±40	-18.7±0.1	S244	cattle *metacarpus III-I sin*. fragment, probably fits together with a *radius sin*.*)* fragment of the same context
**Poz-120015**	5260±40	-23.1±0.1	S246	cattle *phalanx prox*. *anterior/posterior*, disarticulated bone
**Poz-120016**	5340±35	-20.9±0.3	S278	cattle *calcaneus sin*., fits together with another os *tarsale centrale sin*. fragment of the same context
**Poz-119996**	5430±40	-16.1±0.1	S305	cattle *tibia sin*. fragment, disarticulated bone
**Poz-119997**	5330±35	-19.6±0.4	S636	cattle *femur dex*. fragment, fits together with another *tibia dex*. fragment of the same context

*The δ^13^C values cannot be used for paleoecological reconstructions.

### Macrostructure analysis

The investigations aimed to reveal the technologies and manufacturing steps implemented in the production of the Magyaregres ornaments were carried out by using non-destructive and non-invasive methods. The artefacts were examined using a Zeiss Stereo Discovery V8 type zoom stereo microscope and a Zeiss AxioScope A1 polarizing reflected light microscope, at the Archaeometry Laboratory of the Institute of Archaeological Sciences, Eötvös Loránd University, Budapest. Surface traits were analysed through 10–83x magnification under the zoom stereo microscope, and through 25–500x magnification under the polarizing reflected light microscope. The documentation of the traits was completed by a Zeiss AxioCam camera microscope, with the data processed by Zeiss AxioVision software.

Technological examinations were carried out on the following items: seven small cylindrical copper beads, five small tubular spiral coils, the two spectacle spiral pendants and the three spiral bracelets.

### Sampling

All copper bracelets and spectacle spiral copper pendants; as well as, two copper beads and three tubular spiral copper coils were selected for sampling both for lead isotope and chemical elemental compositional analyses.

A Minitor C321 hand drill was used for sampling. The diameter of the PROXXON stainless steel drill bit ranged from 1.0 to 1.6 mm. Prior to sampling, the sample sites were first cleaned thoroughly removing all traces of corrosion. The samples were then extracted, documented and measured. Samples of 6–43 mgs were acquired and sent to the laboratories for further investigation.

### High resolution inductively coupled plasma mass spectrometry

The concentrations of eight elements (Co, Ni, Ag, Sb, Au, Pb, Bi and As) were measured by mean High Resolution Inductively Coupled Plasma Mass Spectrometer [HR-ICP-MS] model Element2 manufactured by Thermo Fisher Scientific (Bremen, Germany) at the Laboratori Nazionali Gran Sasso, Assergi, Italy [72].

Each sample was weighed and dissolved in sonic bath at 60°C, using 10 ml of 10% solution of ultra-pure nitric acid purified with a sub-boiling system (DuoPur, Milestone, Bergamo Italy). After dilution, the samples were measured in Low and Medium Resolution mode in order to minimise the potential risk of isobaric interferences due to polyatomic species generated by the plasma torch. All the operations related the sample handling and the instrumental measurements were performed in a ISO5 clean room to avoid the risk of environmental contamination.

The instrument was calibrated using a single concentration level of standard solution containing certified amounts of the analysed elements. The uncertainties are 20% of the given results.

### Multi-collector inductively coupled plasma mass spectrometry

To investigate the lead isotope ratios the drilled burrs (0.5–10 mg) were dissolved in hot triply distilled concentrated nitric acid in sealed PTFE vessels. As described in Villa [73], the dissolved lead was purified using the SrSpec™ resin (EIChroM Industries) [74]. About 100 ml of resin is filled in a 3 mm diameter hand-made PTFE column. The height to width ratio is approximately four. The sample solution is loaded in 0.5 ml 1M HNO_3_, 1.5 ml of which is also used to wash out the matrix metals, while Pb is very strongly retained on the resin. Pb is then eluted with 1 ml 0.01 M HCl and is ready for analysis.

The measurements of lead isotope ratios were performed using a Thermo Scientific Neptune Multi-collector Inductively Coupled Plasma Mass Spectrometer [MC-ICP-MS] instrument at the Institute of Geology, University of Bern, Switzerland. This instrument is a dedicated tool for highly precise determination of lead isotope ratios, equipped with a double-focusing mass spectrometer of Nier-Johnson geometry. The Faraday collector array allows the simultaneous acquisition of masses 202 to 209. The sample introduction system consisted of an auto-aspirating low-flow (50 ml min^–1^) Apex dissolving nebulizer (ESI Scientific, Omaha, NE, USA) mounted on to a combined cyclonic/double-pass spray chamber made of quartz glass. Potential isobaric interference of ^204^Hg on ^204^Pb was controlled and, if necessary, corrected for by monitoring the ^202^Hg signal. Hydride formation (PbH) was monitored on mass 209 and never detected. Mass fractionation was monitored by adding a small quantity of Tl, which has a known ^203^Tl/^205^Tl ratio, is fractionated by the same mechanism as Pb and does not interfere with Pb isotope measurements [75]. Typical in-run relative uncertainties (2 SE of the mean) on ^206^Pb/^204^Pb, ^207^Pb/^204^Pb, and ^208^Pb/^204^Pb isotope ratios ranged between 0.004 and 0.02%. The measurement accuracy was controlled with frequent measurements of the NIST SRM 981 standard reference material interspersed with the sample measurements. The measured isotopic composition for SRM 981 was indistinguishable from the certified value and the recent, more precise literature measurements [76], so that no adjustment of the measured ratios was necessary. The external reproducibility on the SRM 981 reference material over the measuring period of several days amounted to ± 0.01% (2σ), very similar to the individual in-run precision on unknown samples.

## Results

### AMS dating & Bayesian modelling

**Fig 10:**
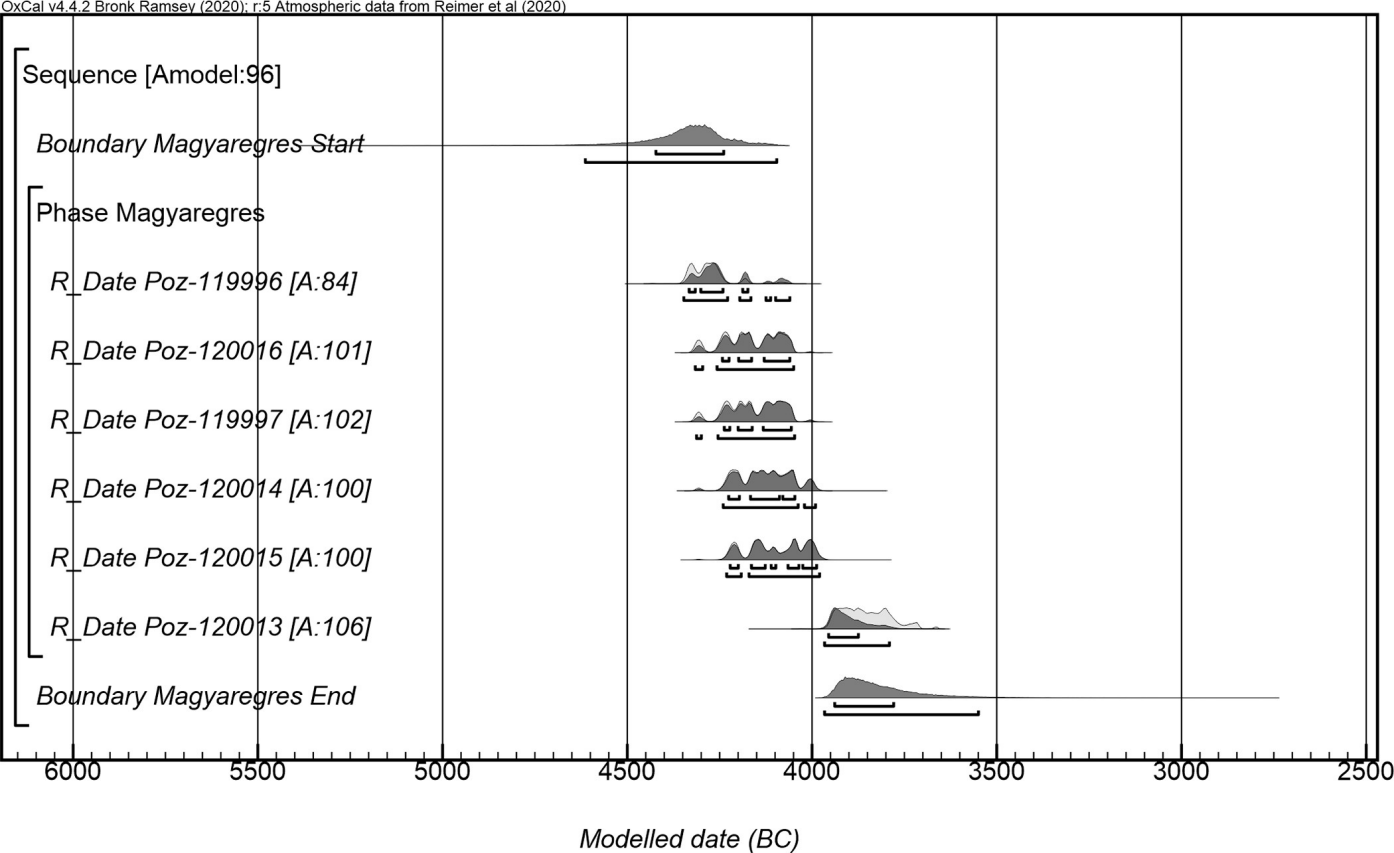
Probability distributions of radiocarbon dates from the Copper Age settlement of Magyaregres-Varga-Bonyi ároktól keletre.

Since there was no superposition between the features containing archaeological material selected for sampling, the radiocarbon data were arranged in a single *Phase* within a *Sequence* (S3 File). According to the Bayesian modelling the establishment of the settlement can be estimated to *4425 (68*.*3%) 4235 cal BCE*, and after *295 (68*.*3%) 445 years* of it being occupied it was then abandoned around *3940 (68*.*3%) 3775 cal BCE* (Fig 10).

### Manufacturing technologies

On the majority of the Magyaregres copper ornaments the technological traits were clearly identifiable under the microscope. The identification and the documentation of these traits made it possible to reconstruct certain steps–or in some cases–the entire process of manufacture.

**Fig 11:**
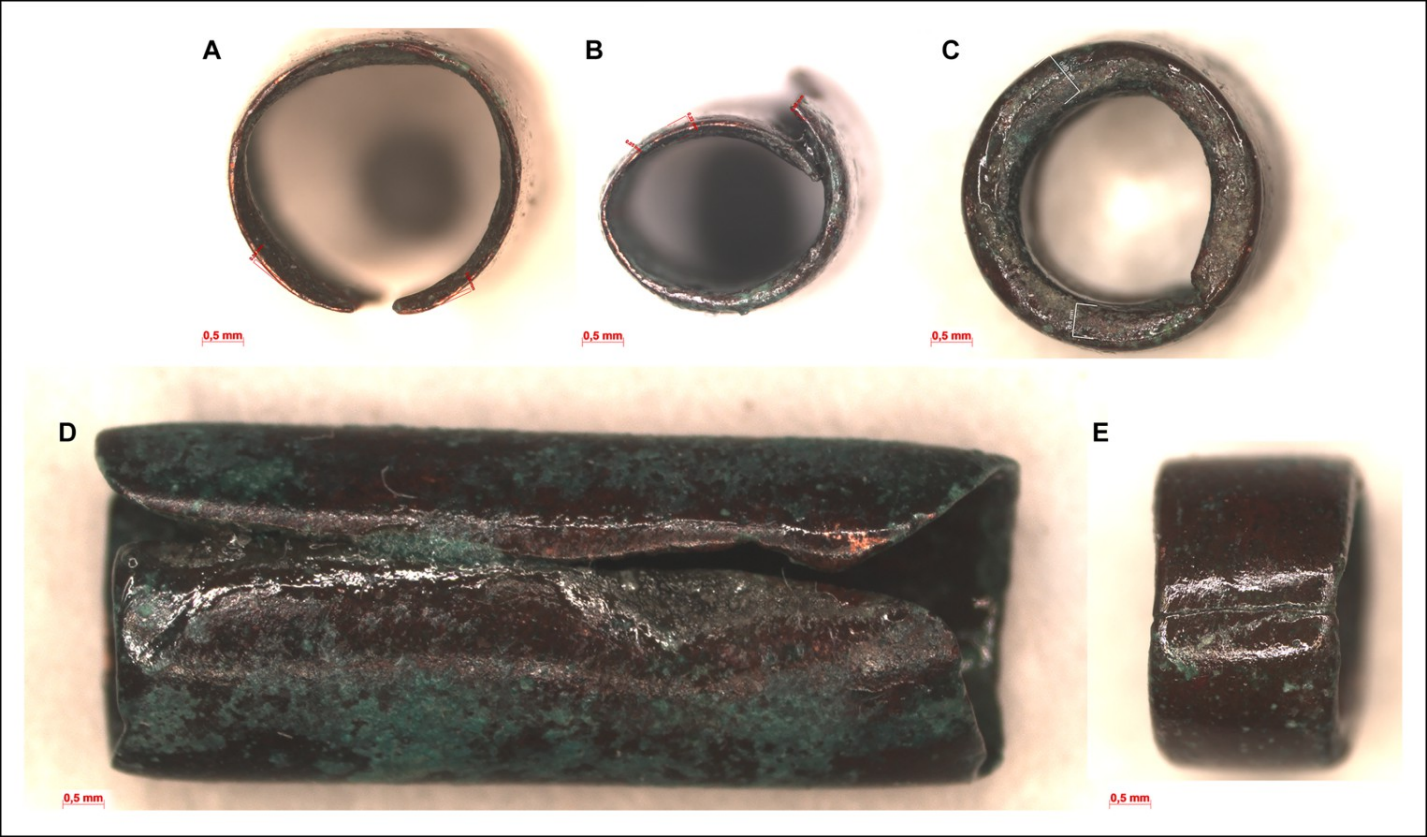
Micrographs showing the macrostructure of cylindrical beads. Photo: Eszter Horváth. (A–C) Plan view of some selected copper beads showing differences in their wall-thickness and quality of the joints (Ő.2019.8.1.283.). (D–E) Side view of some selected copper beads showing differences in their length and quality of the joints.

The cylindrical copper beads show similarities in their manufacture, but their size varies. Along the side of the beads runs a joint indicating that the ornaments were rolled of a copper sheet probably around a cylindrical core of some kind. The quality of the joints ranges from flush through overlapping to gaping. While the short ends of the beads were cut straight, the long, rolled edges were left uneven (Fig 11). Toolmarks were not detected on these edges. Given the malleability of high purity copper, cylindrical beads were probably sliced off from a longer piece of a tube including the core inside, or the copper sheet was cut into small individual rectangles before it was rolled up into a tubular shape. The irregular thickness of the beads’ wall indicates that the sheet was shaped by hammering before it was cut.

**Fig 12:**
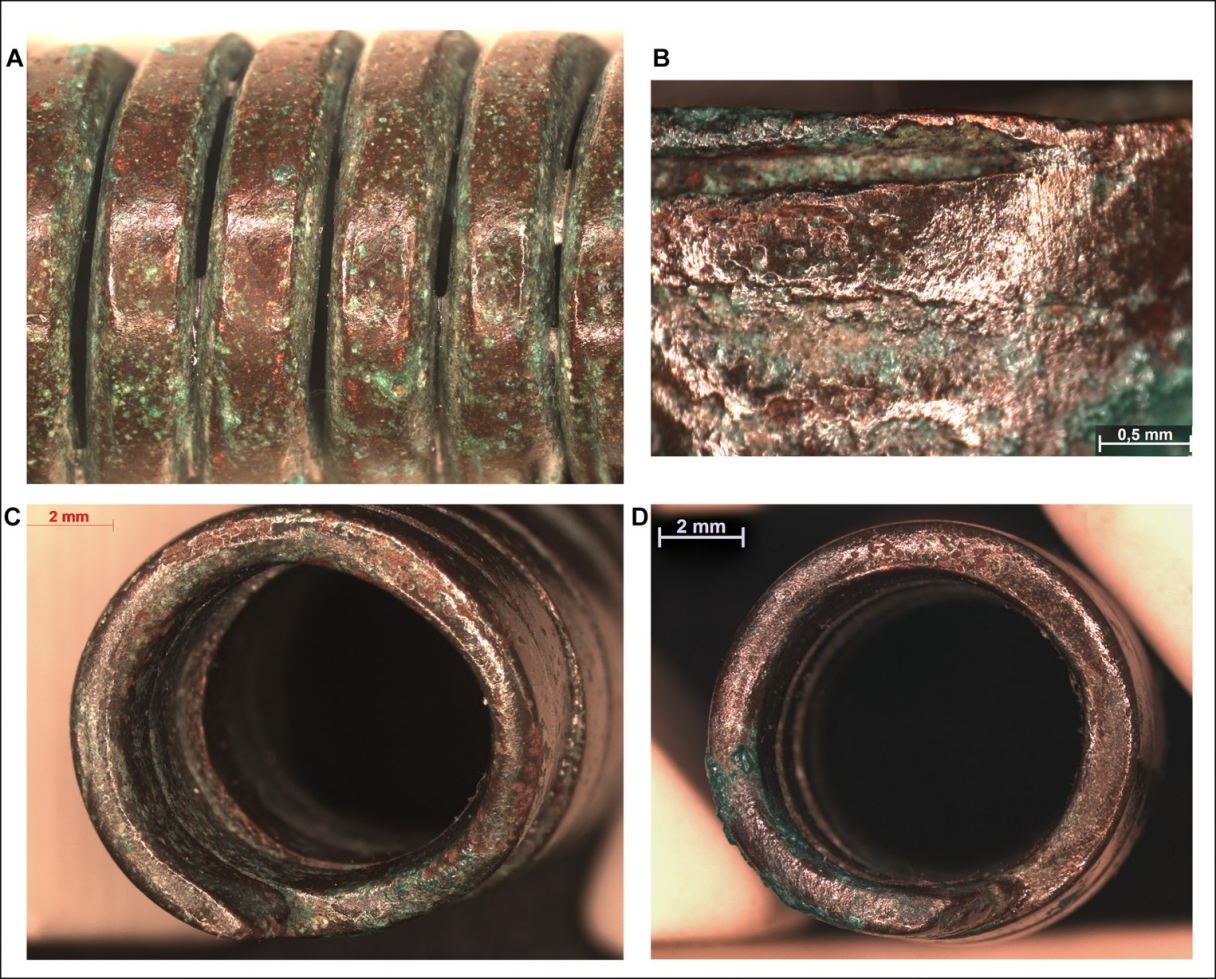
Micrographs showing the macrostructure of tubular spiral coils. Photo: Eszter Horváth. (A) Coiled copper strip with trapezoidal cross-section (Ő.2019.8.1.22.). (B) Short indentation at the edge of a spiral coil (Ő.2019.8.1.179.). (C–D) Plan view of some selected spiral coils showing uniformity in their wall-thickness and diameter (C. Ő.2019.8.1.51., D. Ő.2019.8.1.179.).

The cylindrical body of the tubular spiral coils was created by coiling a piece of copper strip (with trapezoidal cross-section) lengthways (Fig 12A). On the exterior surfaces of the coils–apart from a couple of shorter, scattered indentations–there is no clear evidence for any laminations or joints (Fig 12B). Therefore, it is likely that the 2 mm wide and 1 mm thick coils were made of a single copper strip, probably cut from a larger sheet. Given that all the tubular spiral coils of the hoard had very similar interior diameters, it is feasible to suggest that individual copper strips were coiled around a hard cylindrical core before the items were cut (Fig 12C and 12D).

**Fig 13:**
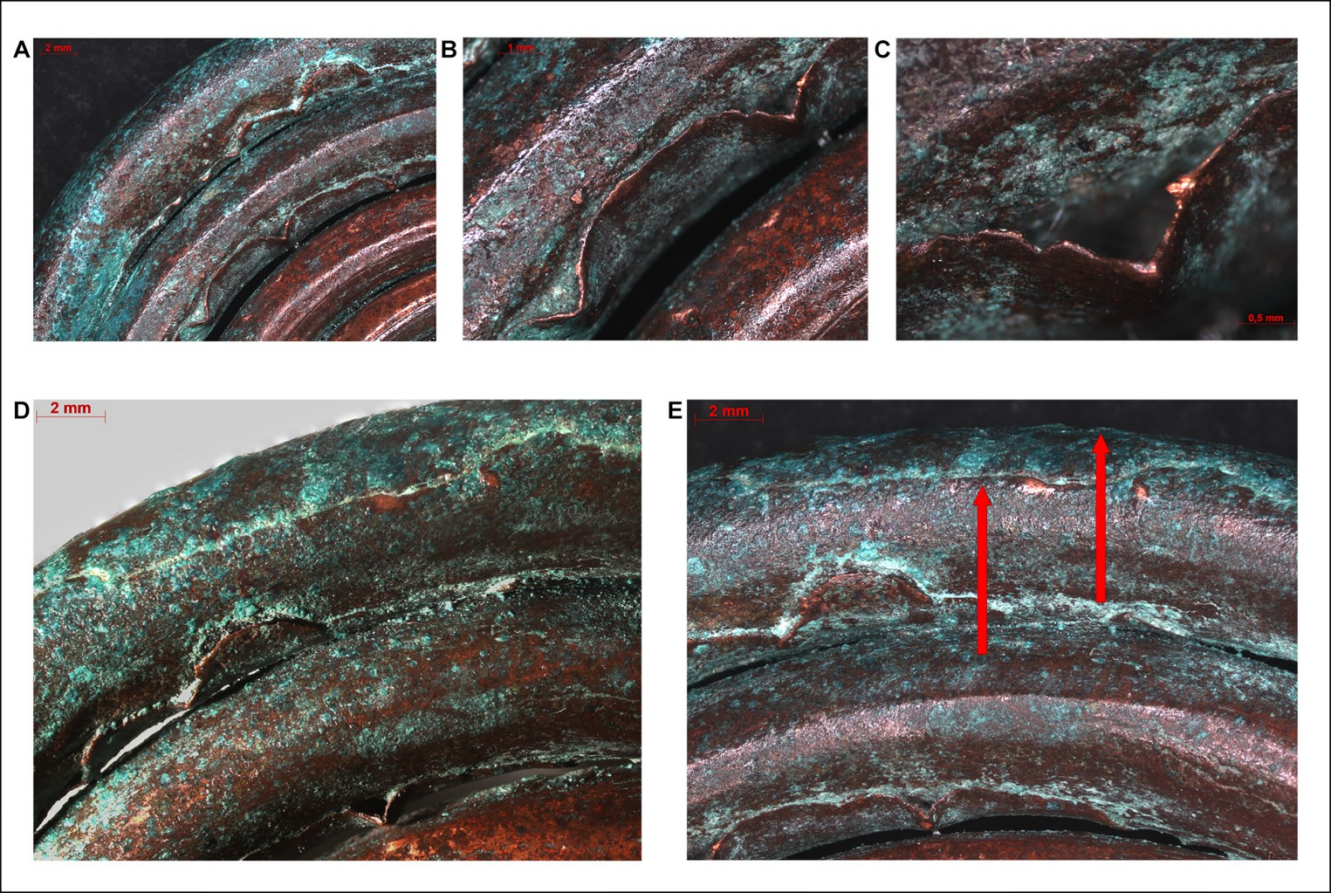
Micrographs showing the macrostructure of the spectacle spiral pendants. Photo: Eszter Horváth. (A–C) The outer edges of the laminated and rolled copper sheets were creased due to the coiling (Ő.2019.8.1.182). (D–E) The outer edges of the laminated and rolled copper sheets shifted a few millimetres off the central axis of the coil (Ő.2019.8.1.182). Red arrows are showing the final, cover sheet with edges facing each other (running in opposite directions).

The surface of the spectacle spiral pendants has shown neither the characteristic dendritic structures associated with casting, nor any seams indicating that these objects were cast. Instead, the pendants had a clearly identifiable laminate structure: stacked copper sheets rolled up lengthways before being coiled into a spectacle-shaped ornament, finishing in two, disc-like elements. The long, joining edges of the copper sheets were uneven in places–partly due to corrosion, partly that the excess material gathered into folds and creases along the inner sides of the coils. The thickness of the copper sheet at these places could be as much as 50 μm (Fig 13A and 13C).

However, the weight of the pendants indicates that the objects were certainly not hollow inside, therefore a tubular structure was unlikely. Upon closer inspection, it became evident that the body of the pendants were constructed by stacking and rolling up several copper sheets (the exact number is unknown), before coiling the two ends into a spiral disc. The length of the individual sheets remains unclear. Due to the coiling, the long edges of the laminated sheets do not align, and can shift a few millimetres off the central axis (Fig 13D). In a single instance it was possible to document two edges facing each other (running in opposite directions) without any joint, which indicates the use of a shorter final layer (Fig 13E).

**Fig 14:**
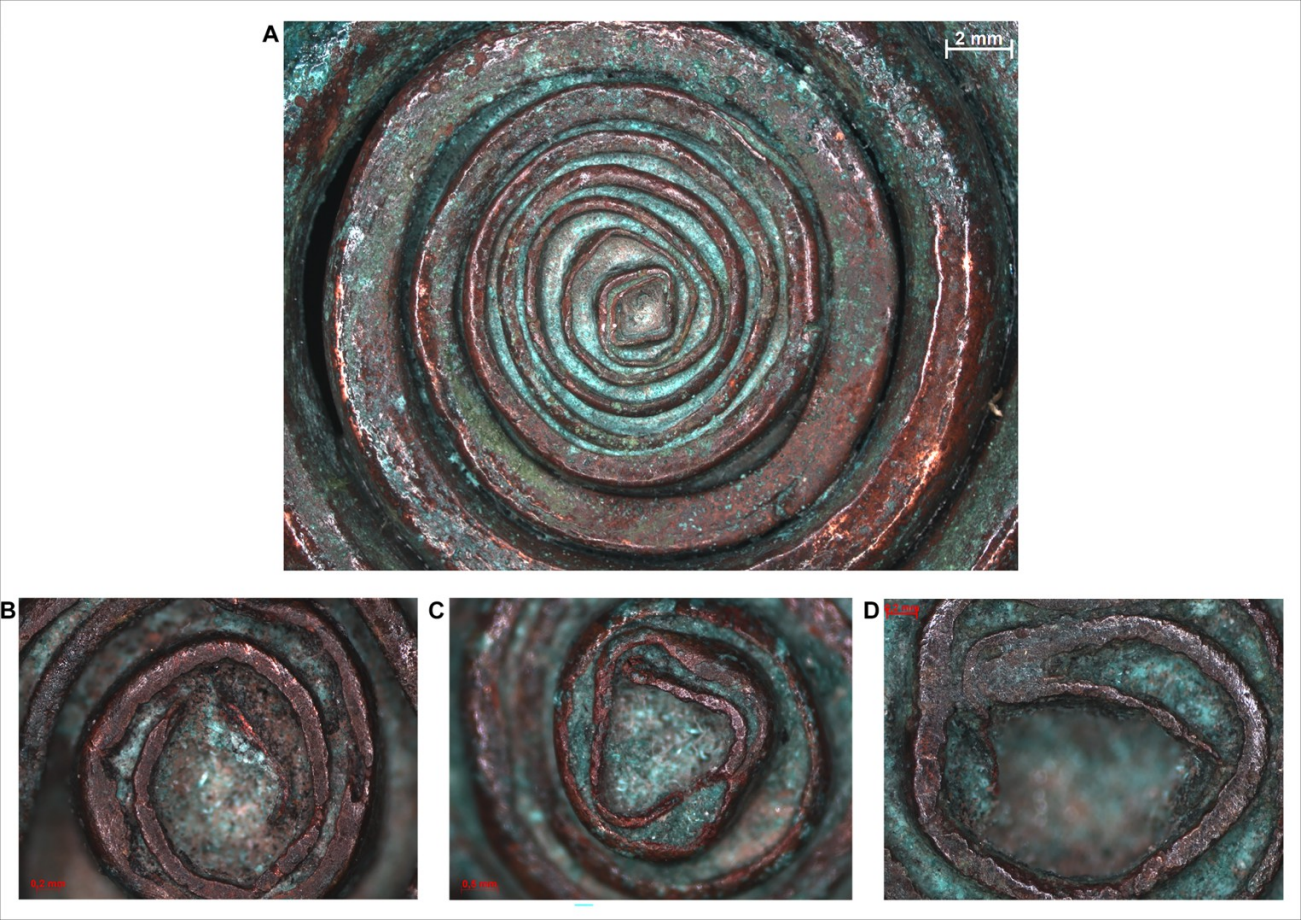
Micrographs showing the macrostructure of the spectacle spiral pendants. Photo: Eszter Horváth. (A) Varying thickness and cross-section on the inner segment of the spiral coils on one of the spectacle spiral pendants (Ő.2019.8.1.183.). (B–D) Inner segments of the spiral coils with oblong cross-section (B-C. Ő.2019.8.1.182., D. Ő.2019.8.1.183).

The manufacturing steps involved in the construction of the pendants can be reconstructed as the following: it is assumed that copper sheets or strips used in the process were of different lengths and widths, indicated by the coils becoming thinner towards the centre of the coiled discs. A possible explanation could be that the length of the copper strips placed on top of each other decreased towards the centre, resulting in an ornament structure that is the thickest in the middle (i.e. around the connecting arch segment of the pendant), and thinnest at the ends (i.e. in the centres of the two spiral discs). The shorter edges of the copper sheets stacked on top of each other could not be observed, which suggests that the entire body of the pendant was covered by a larger and broader final layer (as an outer shell) hiding these edges. The process of coiling must have begun at the ends, preserving the original angular shape of the rolled strip, making the coiled discs to protrude slightly in the centre–the 8–10 innermost coils are rectangular in cross-section (Fig 14A), which presumably consisted of only one or maximum two layers of copper sheets (Fig 14B). The coiling process must have required a considerable force, due to which the coils became rounded, especially towards the middle segments of the ornament. It is possible that the copper sheets were annealed during this process in order to avoid cracking, although such activity did not leave any microscopically detectable traces on the surface of the artefact.

**Fig 15:**
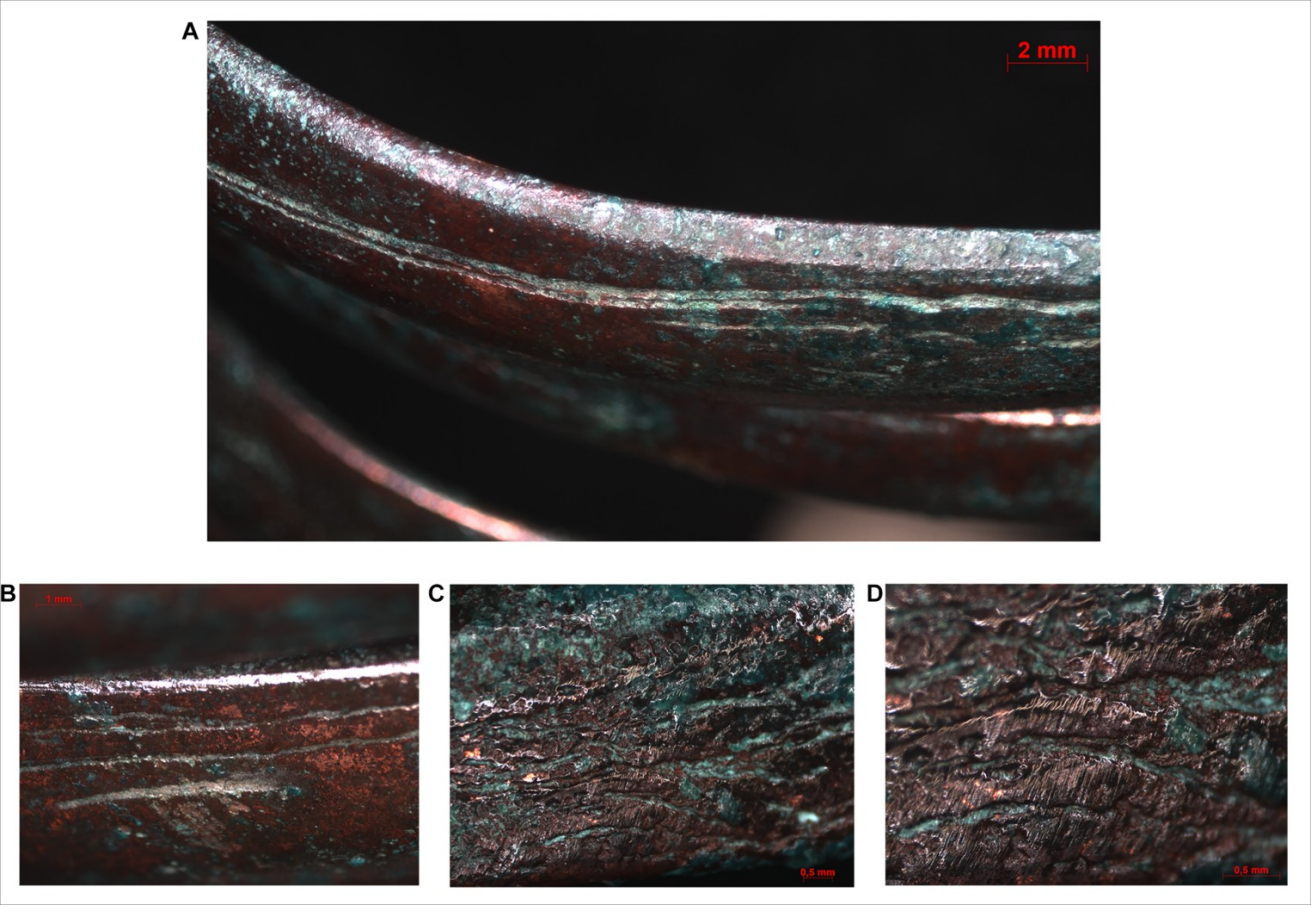
Micrographs showing the macrostructure of spiral bracelets. Photo: Eszter Horváth. (A–D) Parallel lines visible on the surface of the spiral coils suggest a laminate structure (A–B. Ő.2019.8.1.244., C–D. Ő.2019.8.1.243.).

Similar to the spectacle spiral pendants, the surfaces of the spiral bracelets do not imply casting, and the above described laminate structure could be observed here as well. In these cases, the edges of the overlapping sheets are clearly visible under the microscope, running parallel to each other in a distance of 0.5–1 mm along the direction of the coils (Figs 14A–14C and 15A–15C).

**Fig 16:**
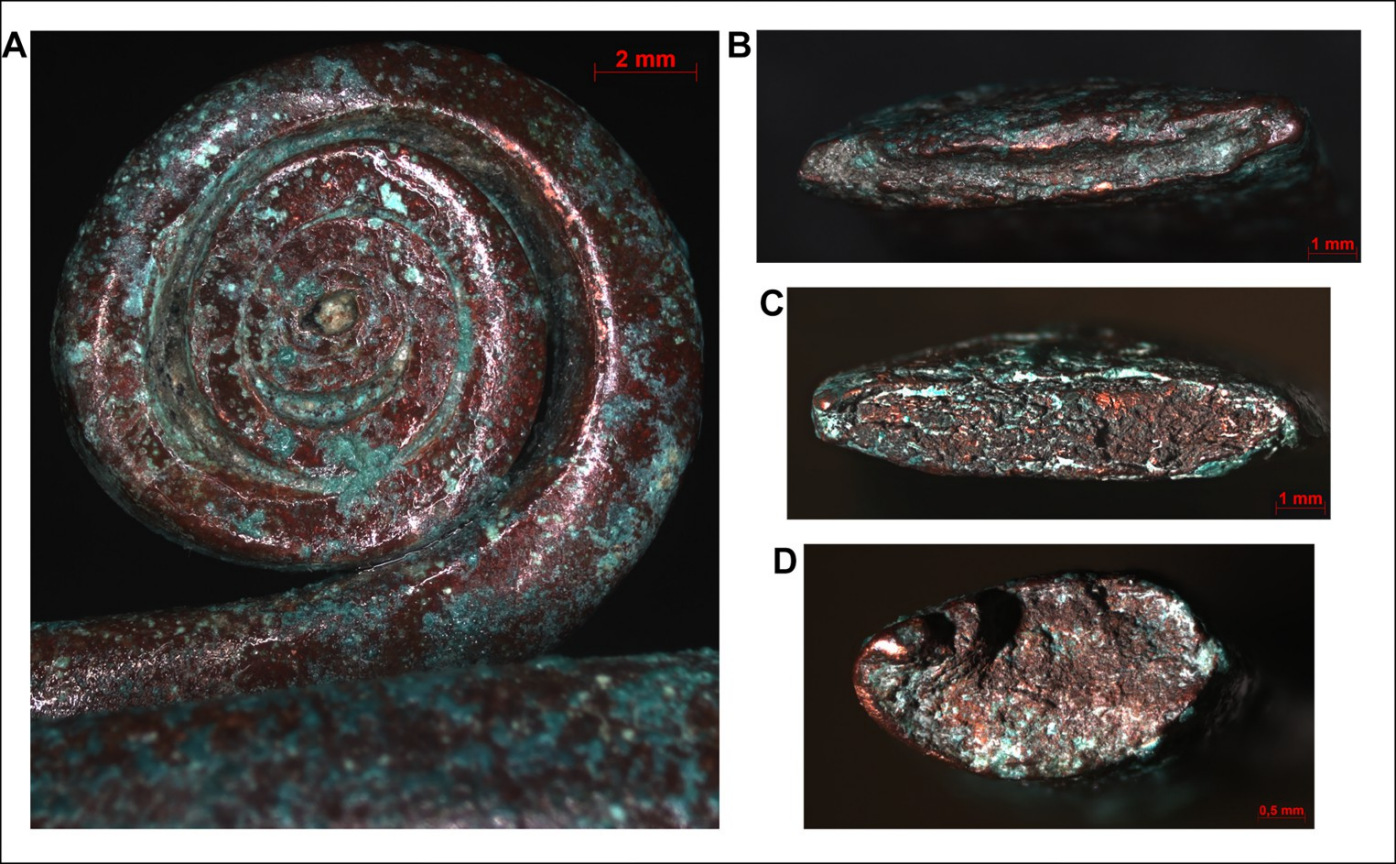
Micrographs showing the macrostructure of spiral bracelets. Photo: Eszter Horváth. (A) Disk-like finial of the bracelet cut in half (Ő.2019.8.1.145.). (B) Cut end of the same segment of the bracelet cut in half showing its lentoid cross-section and laminate structure (Ő.2019.8.1.145.). (C–D) Cut and broken ends of the other segment of the bracelet cut in half showing its lentoid cross-section and laminate structure (Ő.2019.8.1.244.).

**Fig 17:**
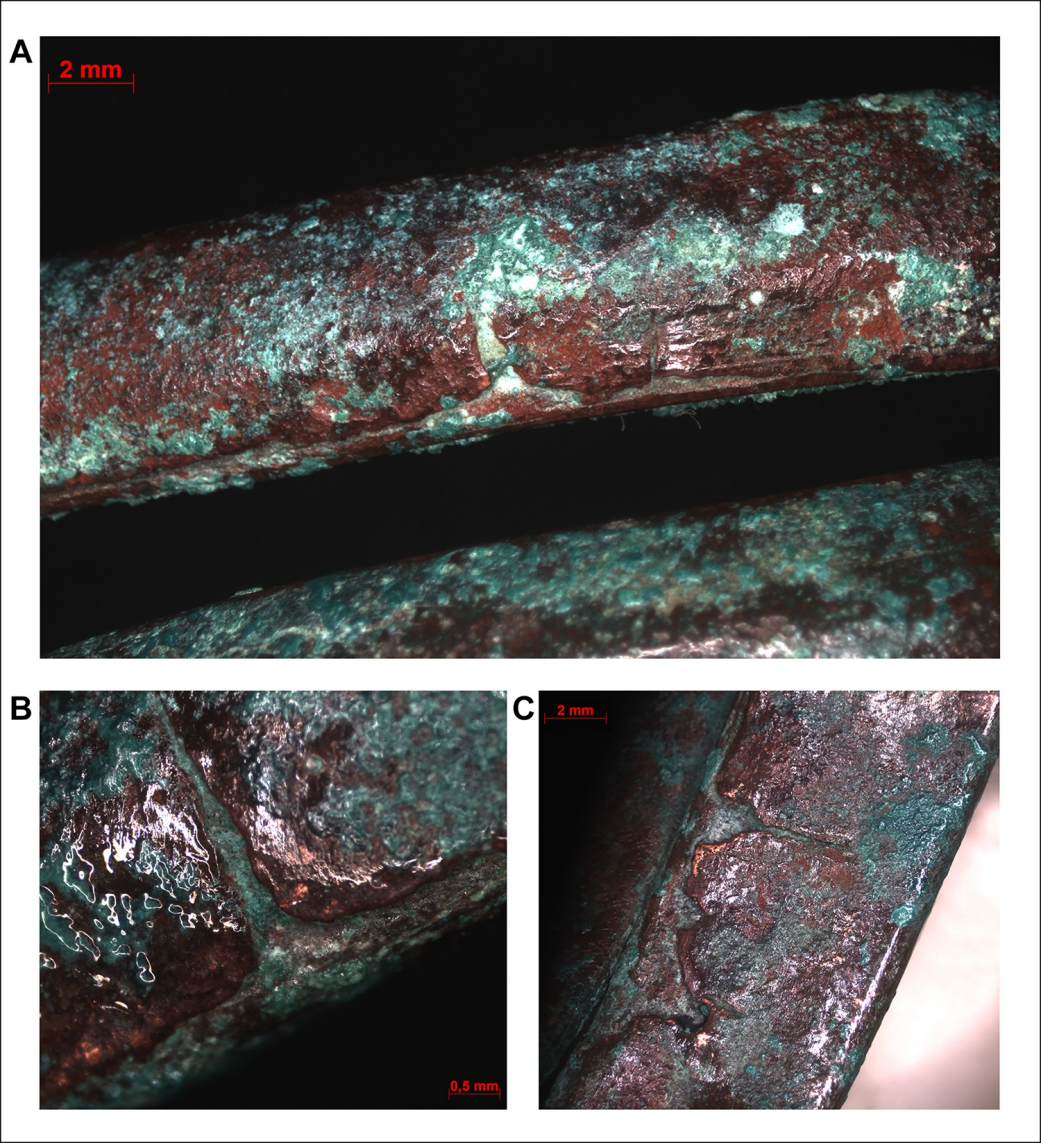
Micrographs showing the macrostructure of spiral bracelets. Photo: Eszter Horváth. (A–B) Cracks on the outer surface of the spiral coil segment of the intact bracelet (Ő.2019.8.1.243.). (C) Cracks on the inner surface of the spiral coil segments of the bracelet cut in half (Ő.2019.8.1.145.).

The stacking of copper sheets before shaping them into cylindrical elements can also be observed in the case of the bracelets however, a key difference here is in the technological steps involved in the achievement the final product. One possibility is that the stacked and overlapping copper sheets were rolled up–similar to the spectacle spiral pendants–and then flattened by hammering or simply hammered into a mould. The other option is that the long edges of the stacked sheets were folded over into thin bands and then the bulk of the material was hammered towards the centre of the coils, making it thicker along the middle segment and thinner towards the edges (Figs 15 and 16). The long, straight strip was then coiled and hammered around a smooth, cylindrical core of some kind, creating a flat surface on the bracelet’s interior, providing a more comfortable wear. The outermost copper strips show minor cracking in some places, probably due to the material being stretched during the coiling process (Fig 17). In order to prevent the formation of cracks, the ornament was probably annealed, although this left no microscopically detectable traces on the surface of the artefact.

### Trace elemental characterisation

**Fig 18:**
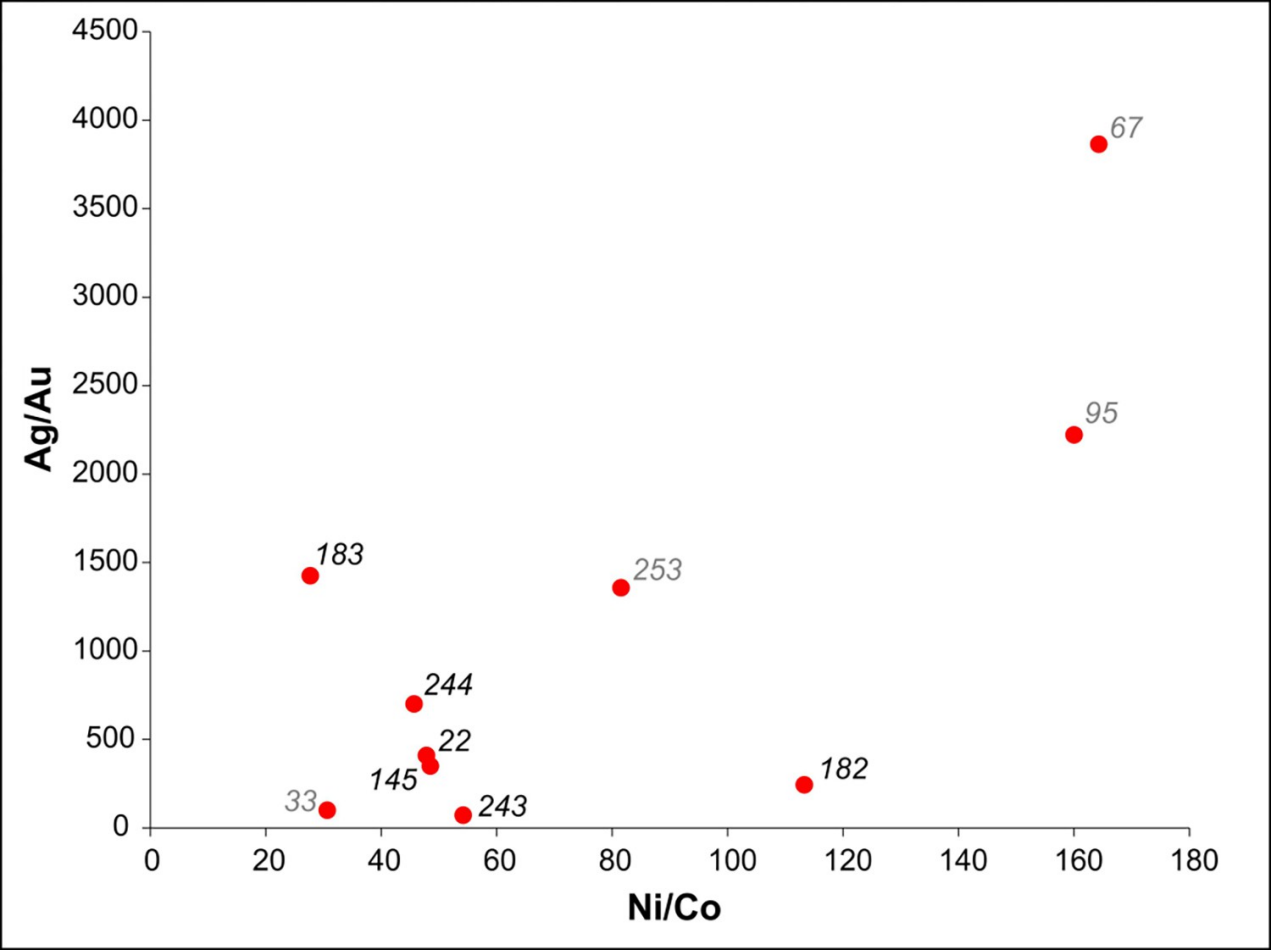
The silver/gold (Ag/Au) ratio versus the nickel/copper (Ni/Co) ratio.

The preliminary X-ray fluorescence analysis of eight selected objects has shown that the items were made of high purity copper (99.6–99.9% Cu) [65]. The trace elements were quantified by HR-ICP-MS method (Table 2). The analysed trace elements were chosen for their diagnostic potential in fingerprinting ore sources, as they and their ratios have predictable regularities in their metallurgical behaviour. The addition of gold to copper ore is not probable, therefore the vertical axis represents true ore heterogeneity in the Ag/Au ratio against the Ni/Co ratio. Similarly, changing the nickel/copper (Ni/Co) ratio only became possible in the 19^th^ century, with the metallurgical achievements of the Industrial Revolution [77]. The dispersion of points in the Ag/Au ratio versus Ni/Co ratio diagram is a robust indicator of source heterogeneity (Fig 18).

**Table 2:** Trace element concentrations (in ppm) of copper objects from the Magyaregres hoard.

Inv. no.	Artefact type	Co	Ni	Ag	Au	Sb	As	Bi	Pb	Ni/Co	Ag/Au	Sb/As	Bi/Sb	As/Sb	Ag/Sb
**Ő.2019.8.1.22.**	Tubular spiral coil	0.23	11	90	0.22	23	1.9	8.4	3.2	47.8	409	12.1	0.37	0.08	3.9
**Ő.2019.8.1.33.**	Cylindrical bead	0.72	22	23	0.23	1.7	0.26	2.7	23	30.6	100	6.5	1.59	0.16	13.5
**Ő.2019.8.1.67.**	Tubular spiral coil	0.14	23	541	0.14	3.3	2.4	1.0	16	164.3	3864	1.4	0.3	0.73	163.9
**Ő.2019.8.1.95.**	Tubular spiral coil	0.15	24	400	0.18	3.8	1.9	1.1	20	160.0	2222	2.0	0.29	0.5	105.3
**Ő.2019.8.1.145.**	Spiral bracelet	0.33	16	14	0.04	0.76	2.6	0.26	5.3	48.5	350	0.3	0.34	3.42	18.4
**Ő.2019.8.1.182.**	Spectacle spiral pendant	0.15	17	44	0.18	5.1	1.6	1.4	4.8	113.3	244	3.2	0.27	0.31	8.6
**Ő.2019.8.1.183.**	Spectacle spiral pendant	0.65	18	242	0.17	5.9	0.65	1.6	5.8	27.7	1424	9.1	0.27	0.11	41.0
**Ő.2019.8.1.243.**	Spiral bracelet	0.24	13	10	0.14	0.32	0.31	0.24	16	54.2	71	1.0	0.75	0.97	31.3
**Ő.2019.8.1.244.**	Spiral bracelet	0.35	16	14	0.02	0.74	0.31	0.26	4.9	45.7	700	2.4	0.35	0.42	18.9
**Ő.2019.8.1.253.**	Cylindrical bead	0.27	22	163	0.12	0.62	0.36	0.16	7.7	81.5	1358	1.7	0.26	0.58	262.9

The concentrations were determined by HR-ICP-MS with a relative measurement uncertainty of 10% for concentrations > 1 ppm, rising to 20% for lower concentrations.

**Fig 19:**
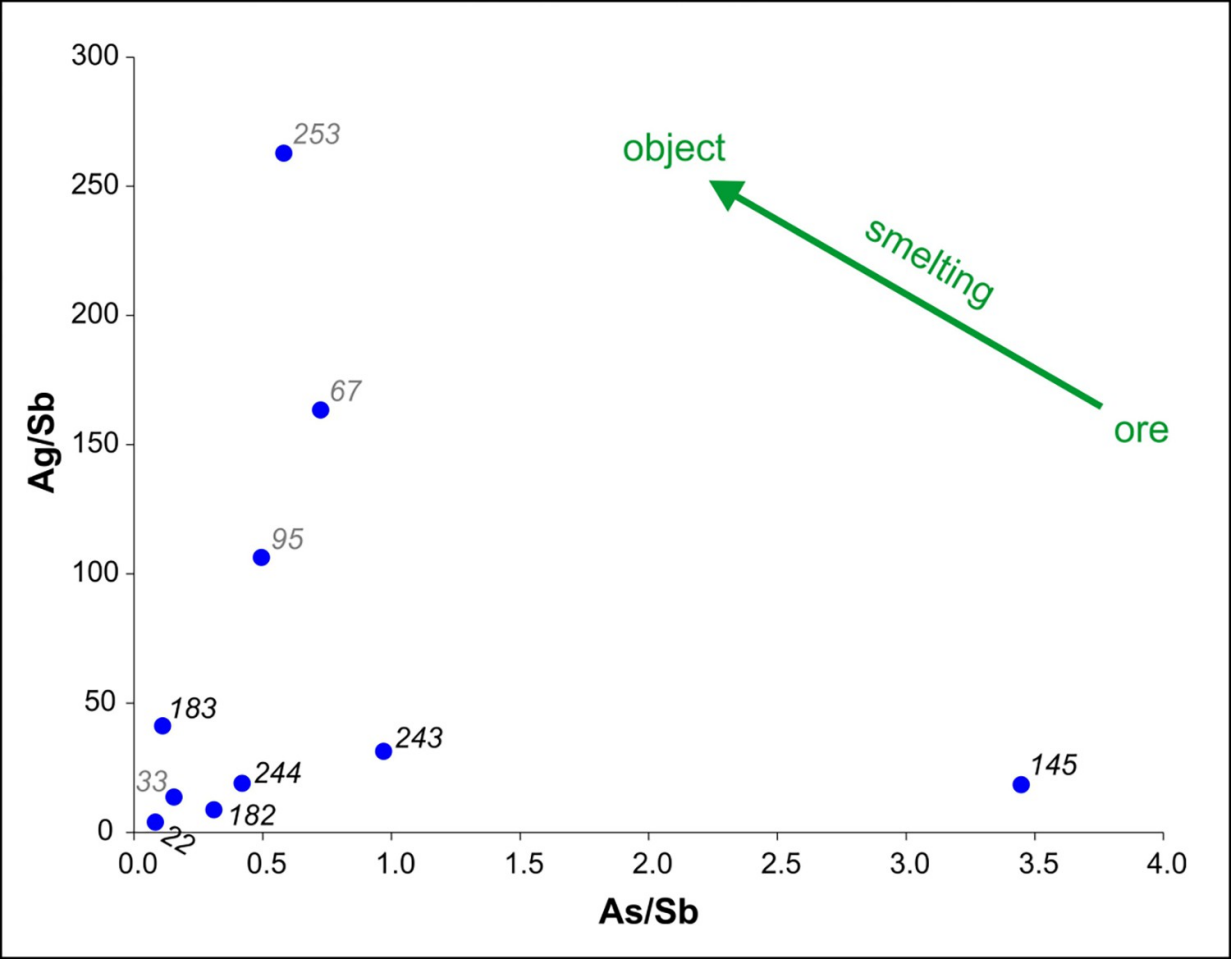
The silver/antimony (Ag/Sb) ratio versus the arsenic/antimony (As/Sb) ratio.

Metallurgical smelting causes arsenic to volatilize, often accompanied by minor antimony volatilization (green arrow on the right in Fig 19) [78,79]. Natural variability of the ore deposits can trend in any direction. The data are not compatible with a single source of metal, whose variations could be explained purely by metallurgical working [79].

**Fig 20:**
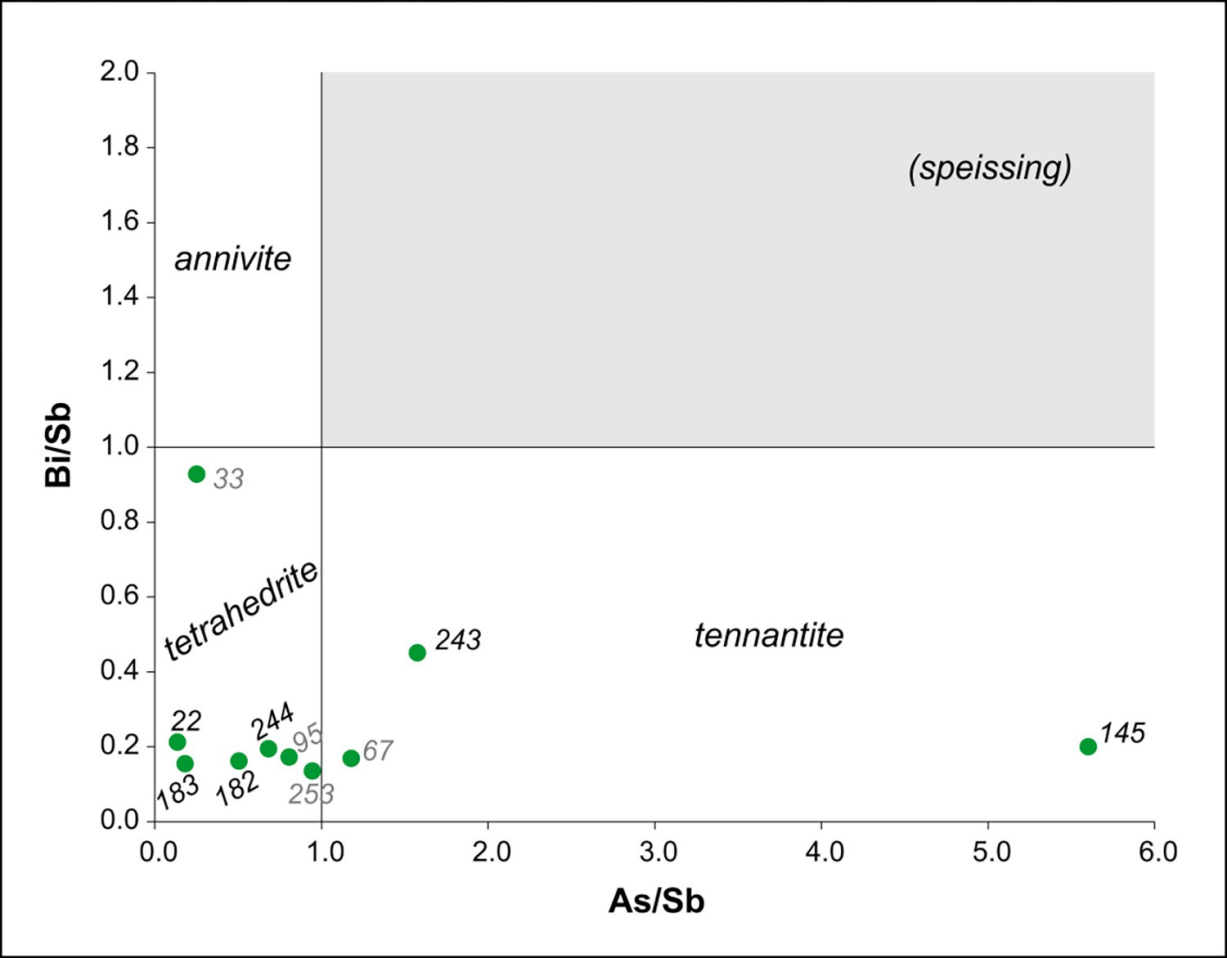
The bismuth/antimony (Bi/Sb) ratio versus the arsenic/antimony (As/Sb) ratio.

Fig 20 is showing the ratios of the elements belonging to group 15 of the periodic table, the so-called pnictogen elements: arsenic (As), antimony (Sb) and bismuth (Bi) have geochemically very similar behaviour and can substitute for each other in a series of ore minerals known collectively as fahlores. The arsenic-rich end-member fahlore is tennantite (in which As > Sb > Bi); the bismuth-rich end-member is annivite (in which Bi > Sb > As); and the antimony-rich end-member is tetrahedrite (in which Sb > As and Bi, usually As > Bi, but not necessarily). The grey-shaded area is "excluded", as it corresponds to no existing pnictogen mineral: it would require As > Sb < Bi, which runs counter geochemical regularity. However, metallurgists of the Early Bronze Age have been consciously adding arsenic-bearing minerals to the mined copper ores to improve the hardness of their products ("speissing") [80]. Speissing can displace the point representing the artefacts towards the exclusion zone if the starting material was annivite-rich. In the present dataset there is no firm evidence for this practice. The overall pattern of the data-points once again confirms the heterogeneity of the ore sources.

### Lead isotope analysis

The outcomes of the lead isotope analyses (Table 3) suggest a pattern of considerable homogeneity. The lead isotope ratios detected on the objects of the hoard fall close to each other considering the diversity and extent of Central European ore fields. Four objects (two beads and two spiral coils) have ^206^Pb/^204^Pb ratios near 18.54 and six are near 18.45 (all bracelets, spectacle spiral pendants and a spiral coils). The differences between the measured ratios can be analytically resolved, but the small difference does not imply a different area of provenance. The hoard samples are certainly not from a single homogeneous ore source, but their raw material very probably originated from different locations of the same mining region. The closeness of the lead isotope compositions and the differences in the trace element signatures can easily be reconciled by viewing the artefacts in the hoard as products of the same source region but not of the same ingot.

**Table 3:** Lead isotope abundance ratios of copper artefacts from the hoard of Magyaregres.

Inv.no.	Artefact type	^206^Pb/^204^Pb	2 SE	^207^Pb/^204^Pb	2 SE	^208^Pb/^204^Pb	2 SE	^207^Pb/^206^Pb	2 SE	^208^Pb/^206^Pb	2 SE
**Ő.2019.8.1.22.**	Tubular spiral coil	18.4355	0.0038	15.6325	0.0031	38.5270	0.0076	0.84796	0.00004	2.08982	0.00007
**Ő.2019.8.1.33.**	Cylindrical bead	18.5476	0.0008	15.6162	0.0008	38.4973	0.0022	0.84195	0.00002	2.07560	0.00006
**Ő.2019.8.1.67.**	Tubular spiral coil	18.5379	0.0020	15.6198	0.0009	38.4954	0.0026	0.84259	0.00006	2.07652	0.00024
**Ő.2019.8.1.95.**	Tubular spiral coil	18.5383	0.0014	15.6200	0.0011	38.4989	0.0041	0.84261	0.00004	2.07679	0.00017
**Ő.2019.8.1.145.**	Spiral bracelet	18.4429	0.0026	15.6373	0.0022	38.5232	0.0054	0.84787	0.00002	2.08879	0.00005
**Ő.2019.8.1.182.**	Spectacle spiral pendant	18.4544	0.0011	15.6428	0.0010	38.5489	0.0026	0.84765	0.00001	2.08888	0.00005
**Ő.2019.8.1.183.**	Spectacle spiral pendant	18.4521	0.0035	15.6427	0.0030	38.5420	0.0127	0.84775	0.00004	2.08918	0.00008
**Ő.2019.8.1.243.**	Spiral bracelet	18.4496	0.0008	15.6363	0.0007	38.5315	0.0028	0.84752	0.00002	2.08844	0.00008
**Ő.2019.8.1.244.**	Spiral bracelet	18.4596	0.0016	15.6438	0.0012	38.5553	0.0032	0.84744	0.00003	2.08858	0.00009
**Ő.2019.8.1.253.**	Cylindrical bead	18.5274	0.0020	15.6387	0.0017	38.5131	0.0045	0.84406	0.00002	2.07866	0.00006

**Fig 21:**
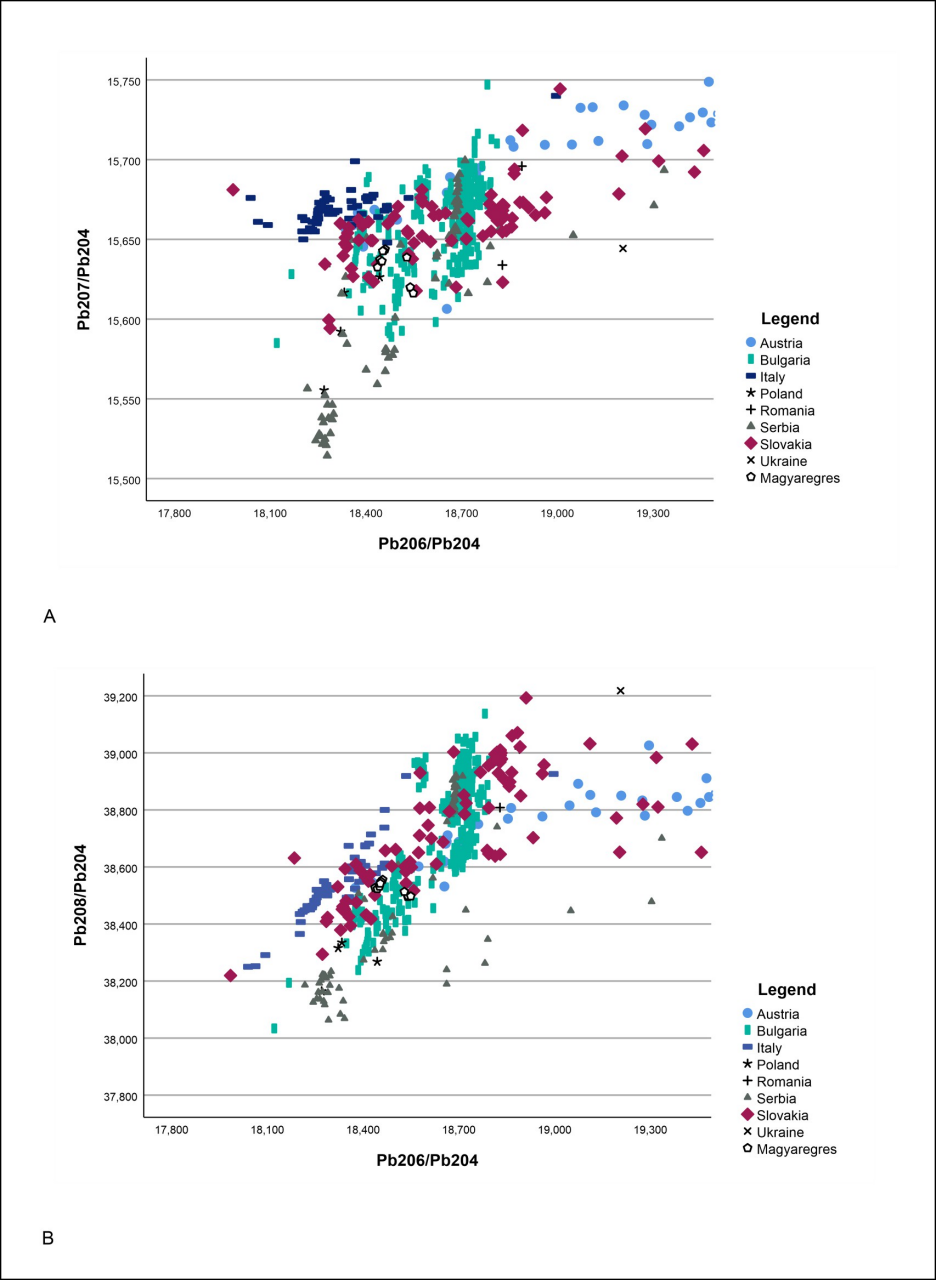
Potential raw material sources of the Magyaregres hoard copper ornaments based on lead isotope analysis. Bivariate plots show the measured lead isotope ratios, compared with the potential ore sources [17–19,32,34,81–84] according to (A) ^206^Pb/^204^Pb vs ^207^Pb/^204^Pb and (B) ^206^Pb/^204^Pb vs ^208^Pb/^204^Pb.

As the next step, the lead isotope signatures of the artefacts were compared with signatures of ore deposits of present-day Bulgaria, Serbia, Romania, Austria, Slovakia, Hungary, Poland, and Italy (Fig 21) [17–20,32,34,81–84]. The signatures produced by the ornaments of the Magyaregres hoard were undoubtedly different from the lead isotope signatures characteristic of the southern Alpines ore sources [83,85], thus these locations could be excluded from the list of potential provenances. The signatures of most copper ores from Slovakia to Bulgaria fall all very close to each other (the geological evolution of the entire area was rather uniform, and so are the ores [17,18]). However, the currently known number of geological sites is low–yet there must have been hundreds, if we extrapolate from what was documented from a relatively small portion of the Alps: small outcrops sufficient to produce a few kilograms of copper occur every few kilometres [83].

Among the charted deposits closest to the Magyaregres hoard, those compatible with the present analyses are Panagyurski (550 km SE), Vratsa (450 km), and the comparatively broad field of ores from Slovakia (e.g., Špania Dolina, 250 km N of Magyaregres) [17,19,34,81]. None of these deposits is uniquely constrained. It must be kept in mind that some mining regions are hardly studied for their lead isotope signature: Romania, Hungary, and the western Balkans [84,86,87].

## Discussion

### The source and composition of raw materials

10 of the 705 copper objects were sampled for lead isotope and chemical compositional analyses. Each of the spectacle spiral pendants and bracelets, three of the 19 spiral coils and two of the 681 beads were analysed. The aim of these examinations was to reconcile the expected scientific results with the conservation of the artefacts. Therefore, after realising that the raw material of the spiral coils and the beads is very similar to the rest of the objects, we decided not to proceed with taking more samples, as it was unlikely that these would reveal further variation in the raw material.

**Fig 22:**
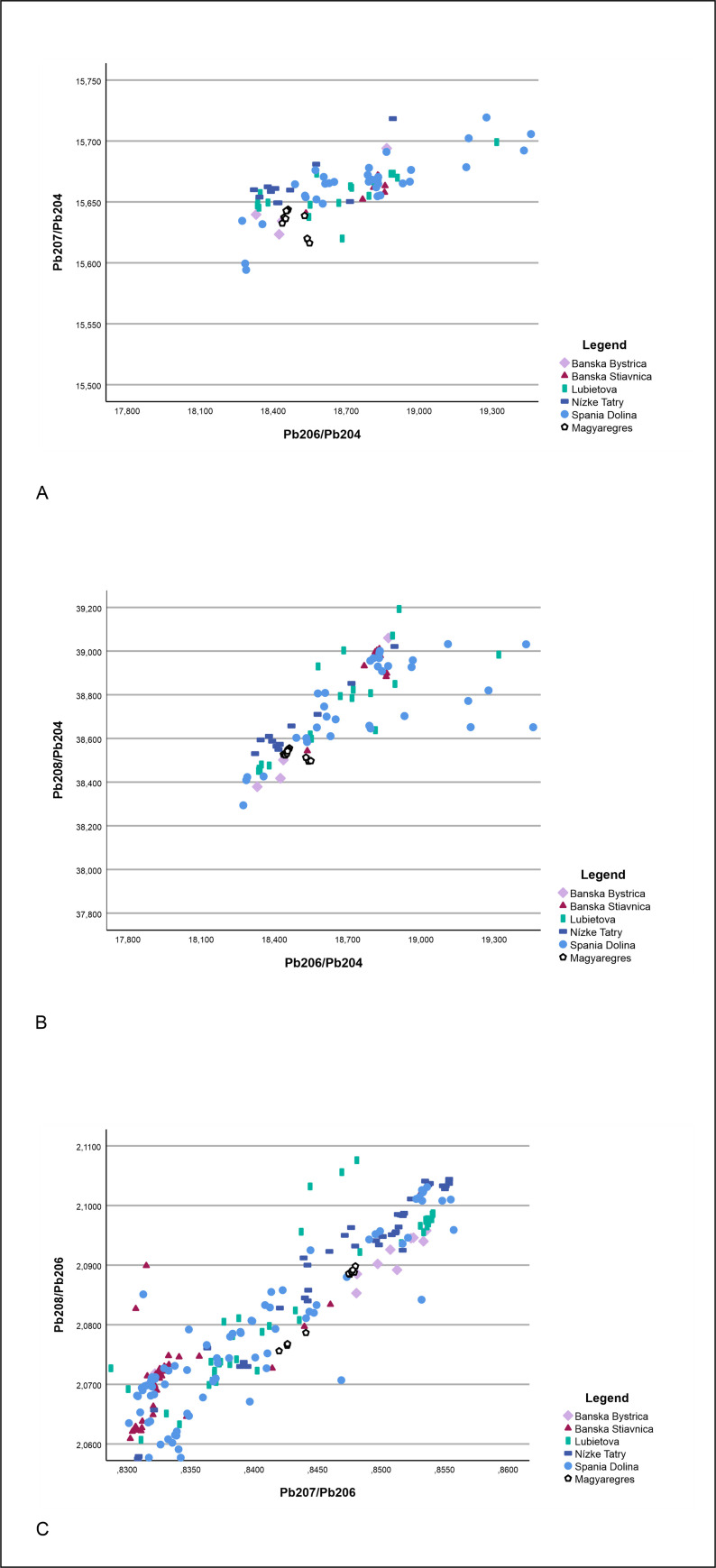
Potential raw material sources of the Magyaregres hoard copper ornaments based on lead isotope analysis. Bivariate plots show the measured lead isotope ratios, compared with the potential ore sources from Slovakia [34] according to (A) ^206^Pb/^204^Pb vs ^207^Pb/^204^Pb, (B) ^206^Pb/^204^Pb vs ^208^Pb/^204^Pb, and (C) ^207^Pb/^206^Pb vs ^208^Pb/^206^Pb.

Trace element analyses have revealed that fahlore copper was used to produce the Magyaregres ornaments; the majority of the artefacts were made of high antimony (Sb) fahlores, while for a smaller number of objects arsenic (As) and bismuth (Bi) fahlores were used. Although, based on the lead isotope ratios, the copper sources of Bulgaria could be included among the potential ore sources, there are no fahlores documented from the region [17,19,20,88]. Therefore, the most likely source of fahlore coppers is located in the Slovakian Ore Mountain region [34]. Here, fahlore copper deposits occur most characteristically around the areas of Špania Dolina and Banska Bystrica in the Hron/Garam Valley [34,38–40]. The ores from this region are even more chemically diverse than their lead isotope compositions [34], however their signatures correspond well with the ratios measured on the objects of the Magyaregres hoard, which supports the assumption that the raw material for the ornaments could have derived from these Slovakian ore sources (Fig 22). From the Banska Štiavnica area, there are several ores with extremely high Bi/Sb contents–however, all these Bi-rich ores have very high ^208^Pb/^204^Pb ratios [34], while the Bi-rich inv. no. Ő.2019.8.1.22. spiral coil from the hoard has the lowest ^208^Pb/^204^Pb, therefore it cannot derive directly from these particularly Bi-rich sources around Banska Štiavnica. However, Marcus Schreiner observed that Bi was present in a small number of Slovakian ores and could also have been present at Špania Dolina (or in the less studied valleys north and south of the Hron/Garam) [34].

Among the Early Copper Age artefacts from Slovakia, items made of fahlores with trace elements of arsenic, antimony, bismuth and silver occur relatively frequently, and most probably originate from Slovakian ore sources. These sources are most likely located around the areas of Ľubietová and Špania Dolina, where (antimony-bearing) fahlore and chalcopyrite dominate among the local ores [34]. The utilisation of these ores is evidenced by the ingot found among the items of the Handlová hoard. The Nógrádmarcal and Handlová-type heavy, copper hammer axes were also made from fahlore coppers [89,90]. These raw materials have long been linked to Slovakian ore sources [89,91–96], supported recently by chemical compositional analyses [34,35]. At the site of Slovenské Pravno in an Epilengyel context, evidence for local metallurgy was found, confirming the utilisation of the nearby Špania Dolina-Piesky tetrahedrite source [34,97]. The prehistoric exploitation of the ore source has been verified by archaeometallurgical and montanarchaeological research, although the exact dating of these activities remains uncertain [39]. The utilisation of fahlores ceased towards the end of the Early Copper Age, then resurfaced and became widely used again during the Early Bronze Age [38]. The utilisation of these copper sources is contemporaneous with the Magyaregres site.

In his work, Marcus Schreiner analysed 330 ore minerals collected over ca. 2000 km^2^ from the Hron/Garam River Valley in central-northern Slovakia [34]. Only a quarter of the ore samples included in the study produced the full range of lead isotope data; for most of the samples, only trace element concentrations were reported. Furthermore, only four elements–among the eight elements which were measured and plotted here–were selected and measured consistently across all samples making it difficult to compare with the Magyaregres ratios. Nevertheless, the Hron/Garam Valley appears to be a possible source for the objects studied here. Isotopic data from this region spans across a very wide Pb isotope composition field (^206^Pb/^204^Pb ratios range from 18.28 to 19.46, and all other Pb isotope ratio exhibit similarly broad ranges), which includes the very compact field defined by the Magyaregres hoard samples. There are about six of Schreiner’s samples, from three separate localities (one from Ľubietova, one from Banska Štiavnica, and approximately four from Špania Dolina) [34], which plot quite close (possibly even within the limit of uncertainty which unfortunately was not included in the publication) to the hoard.

Some of the ore analyses that have been reported so far in the literature show a very similar range, although not identical, perhaps due to the quite compact distribution of the 10 hoard samples. This may be explained by (1) the exact mining site has not yet been discovered or analysed, (2) the ores were extracted from a single mine, but its internal variability (which is well-known from modern mines, even at the cm scale [98]) means that the ores accessible today derive from slightly different levels from the ores accessible 6000 years ago, (3) the ingots used to make the hoard objects were themselves the result of combining neighbouring sources. However, the recycling of already existing objects, as it has been argued in the case of Late Bronze Age oxhide ingots [99], is not relevant prior to copper becoming an industrial commodity. The results of the lead isotope analysis performed on Copper Age items show a great deal of heterogeneity which Ernst Pernicka considers as evidence against large scale recycling at the time [100].

In summary, geographically the Slovakian mining region of Ľubietová and Špania Dolina represents an ideal location for sourcing copper for communities living further downstream on the plains of Transdanubia. Finished products may have been transported by boat down the Hron/Garam and then the Danube before making their way to the region of Magyaregres. Therefore, beyond the matching chemical and isotope signatures detected between ores and artefacts, the logistical advantage represented by the Slovakian ore sources increases the likelihood in the utilisation of these sources over the Balkan ores producing similar signatures.

Formerly, Nándor Kalicz proposed the existence of a metallurgical centre within the territories of the Jordanów/Jordansmühl-Ludanice-Balaton-Lasinja complex, based on the distribution and typological analogues of Balaton-Lasinja metal finds in Transdanubia [21]. Later, primarily on typological grounds, several copper artefacts were associated with the Balaton-Lasinja culture suggesting a link between these objects and the Slovakian ore sources [53,94,101], however, to support this assumption lead isotope analyses have not been carried out on Copper Age copper artefacts from Transdanubia until now.

Lead isotope analyses have not yet been conducted on copper artefacts similar to the types included in the Magyaregres hoard and only a couple of ornaments have undergone chemical compositional analyses. One of these is a fragmented spectacle spiral pendant from Pohořelice, which was produced from copper with a high antimony content, the raw material for which is assumed to be from the mines of Špania Dolina [35]. The Stollhof-type discs and the Malé Leváre-type spectacle spiral ornaments are also considered as made of high antimony copper. Based primarily on the distribution of these objects, the existence of an independent metallurgical centre within the Western Carpathian region associated with the Jordanów/Jordansmühl-Ludanice-Balaton-Lasinja complex has been suggested [36,102–104], supported by chemical compositional examinations [34–36,105]. The copper artefacts discovered at the hilltop settlement of Kotouč-Štramberk in the Czech Republic were crafted of copper sulfides and can be classified into two main groups. The pectoral and the fragment of a Nógrádmarcal-type axe were made of copper containing antimony and silver, while for the flat axes and the spectacle spiral pendant, arsenic copper was used. Therefore, the exploitation of two different copper sources can be presumed [36,46].

A direct relationship between the Magyaregres ornaments and the Rudki-type double spiral pendants from Poland can also be implied both in terms of the raw material and the manufacturing technology [33]. As lead isotope and chemical compositional analyses have shown the Late Copper Age ornaments found at the sites of Kałdus and Przeuszyn were also made of Slovakian copper ores [33].

From a technological point of view, sulphidic ores were already being smelted during the second half of the 5^th^ millennium cal BCE as evidenced by the assemblages found at Gumelniţa and Varna culture sites in Bulgaria [106], which also suggests that people experimented with different ore types even during the early phases of copper metallurgy. Therefore, the smelting of sulphidic ores cannot be considered the next evolutional step in developing metallurgy [107], but could rather be recognised as different trajectories of explorations targeting a range of ore sources and minerals. The emergence of a local metallurgical circle in the Northwestern Carpathians therefore can be regarded as the consequence of experimentation processes based on the exploitation of local fahlore sources and utilising local metallurgical skills and traditions by the period of the Early Copper Age.

### Manufacturing technologies

The *chaîne opératoire* and the manufacturing technologies involved in producing objects of a particular type appear to be highly standardised, and certain technological characteristics were shared across different types of objects of the hoard. Thinner ornaments (such as cylindrical beads and tubular spiral coils) were made of a single copper sheet. Thicker pieces (like bracelets and pendants) were constructed by stacking several copper sheets to create laminate structure which was then rolled or folded and coiled into a spiral shape. The sheets were flattened and shaped by hammering. Standardisation is reflected also by object shapes: cylindrical, tubular and spiral forms occurred.

The coiling technique employed to create large-sized ornaments resulted in rigid, solid pieces. This rigidity certainly needed to be addressed during the coiling process, in order to prevent cracks and fissures. A possible method for making the strips more malleable is annealing by heating either during or at the end of the hammering process but still prior to coiling, before their hardness was restored after cooling. However, the exact way of this possible annealing is invisible on the surface.

The surface observation of the objects only allows us to appreciate the traces of the last intense work phase. To identify the whole manufacturing process further investigations are required to be performed: the internal structure can either be identified by radiographic imaging or the microstructure can be revealed by metallographic analysis.

**Fig 23:**
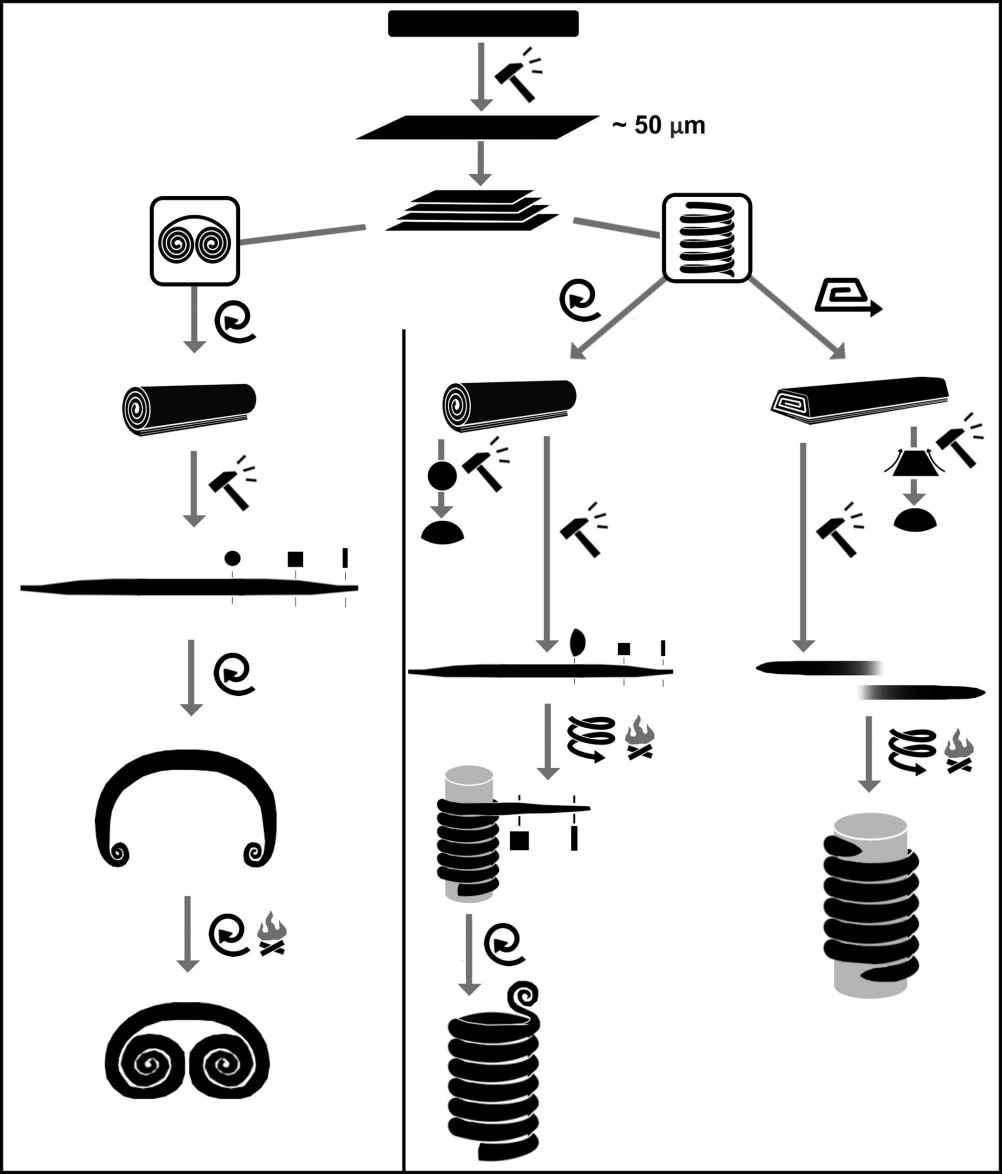
The reconstruction of *chaîne opératoire* of spectacle spiral pendants and spiral bracelets. Drawn by Katalin Sebők.

By examining the spectacle spiral pendants under a microscope, the following manufacturing steps could be reconstructed (Fig 23):

1. hammering (flattening, thinning) of copper sheets or strips of various length, width and thickness (achieved thickness is approx. 50 μm)
2. lamination of copper sheets; broader pieces, served as the outermost layer of the ornament, were placed at the bottom, upon which gradually narrowing sheets were stacked in a symmetrical structure
3. laminated strips were then rolled to create a cylindrical structure (probably around a cylindrical core of some kind), before finishing the overlapping edges
4. in one case, the coil was capped by a shorter final sheet to cover up the irregularities (Fig 13E)
5. the two ends (approx. 200–200 mm sections) of the laminated cylindrical copper coil were hammered to create a square and then towards the ends, an oblong cross section (Fig 14A)
6. the thinned, rectangular ends were coiled into a spiral with a protruding centre (probably also shaped around a core)
7. continuation of the coiling process from each (i.e. opposite) direction, during which the piece might be annealed make it more malleable, and the stretching of the coil easier.

Similarly, the *chaîne opératoire* of spiral bracelets can be reconstructed as the following (Fig 23):

1. hammering (flattening, thinning) of copper sheets or strips of various length, width and thickness (achieved thickness is approx. 50 μm)
2. lamination of copper sheets; broader pieces, served as the outermost layer of the ornament, were placed at the bottom, upon which gradually narrowing sheets were stacked in a symmetrical structure
3. option #1 laminated strips were then rolled to create a cylindrical structure (probably around a cylindrical core of some kind), before finishing the overlapping edges, or
4. option #2 the long edges of the stacked sheets were folded-up in a narrow band
5. option #1 shaping the rolled strips–probably by hammering–to achieve a semi-circular cross section, or
6. option #2 shaping the folded-up band: thinning of the long, folded-up edges, while hammering the bulk of the material towards the central axis of the band
7. case #1 (the bracelet cut in half): the intact end of the ornament was hammered to achieve a square cross section, gradually becoming oblong
8. case #2 (the intact bracelet): the ends of the ornament were shaped round-pointed
9. case #1 (the bracelet cut in half): The ornament was coiled up to form a spiral (in the segments with semi-circular cross section). The regular execution of the ornament suggests that it was coiled around a cylindrical core of some kind.
10. case #2 (the intact bracelet): The method was similar: the ornament was coiled into a spiral shape with the aid of a smooth, cylindrical core.
11. case #1 (the bracelet cut in half): The piece was finished off by coiling its intact end (the segment with rectangular cross section) into a small spiral disc, axis of which is orthogonal to the main axis of the bracelet.

Due to high levels of corrosion affecting the majority of copper artefacts, similar observations concerning the manufacturing steps can rarely be carried out. For the production of the copper bracelets found in the cemetery of Durankulak, Bulgaria, two different technologies were employed: casting and hammering. Research has shown chronological differences and different raw material sources associated with the two technologies [108].

Despite their slightly younger dating, the Late Copper Age so-called Rudki-type double spiral ornaments from Poland and the copper finds of the Magyaregres hoard can be linked in terms of their technology and the provenance of raw materials [33]. The Rudki-type spiral ornaments were initially considered as solid metal artefacts, where several copper rods were mechanically combined lengthways using cramped joints, prior to the coiling process [33]. Comparing the images published on the Rudki ornaments with the observations of the better preserved Magyaregres assemblage described above, it is feasible to assume that these items also had a laminate structure before they were shaped into double spirals. Given the high levels of corrosion detected on the Rudki pieces, this might be the reason why certain deteriorated sections were mistakenly identified as joints, as the outer sheets peeled and disintegrated substantially in certain places.

Nevertheless, the question remains: where could have the metallurgical workshop producing such objects been located? Hoards containing copper ingots were documented not only in close proximity to the raw material sources in Slovakia (e.g., Handlová), but further afield (e.g., Nedakonice) as well [91,96]. The presence of ingots and crucibles suggests that the manufacture of copper ornaments was not limited to the near vicinity of ore sources but that semi-finished products were used to produce ornaments in more distant territories [35]. Crucibles are known from the eastern, western and southern regions of Austria (e.g. Bisamberg-Oberpullendorf, Keutschacher See, Brixlegg) and from the Czech Republic (e.g., Makotřasy) dating to the same period [32,109–112], however, so far from the territory of Hungary these items are absent. The crucible of Brixlegg represents a prime example that although these artefacts could serve as evidence for the existence of local technological knowledge in metal smelting, they do not necessarily prove the exploitation of local ore sources [32].

Similarly, there is no evidence for local metallurgical activities either at the settlement of Magyaregres, or in Transdanubia as a whole during the Early Copper Age, not even in those instances when copper artefacts were found at contemporaneous sites. Zsuzsanna M. Virág while excavating the site of Zalavár-Basasziget came to the conclusion that copper objects were not made locally, but were imported to the settlement [53]. Likewise, in the case of the artefacts of the Magyaregres hoard could have found their way into Transdanubia through a series of social networks as finished products. Crucibles indicating the existence of local metallurgy only appear during the subsequent chronological phase of the Furchenstich culture in Transdanubia [21,113].

In his recent comprehensive study on Bronze Age metallurgy, Maikel H. G. Kuijpers makes a distinction between the technological knowledge possessed by mining communities and the skills of metalworkers. The craftspeople, producing artefacts from ingots could be geographically far removed from the raw material sources and were not necessarily aware of the details of how ingots were produced and from what they originated of [114]. This distinction between different spheres of technological knowledge could be reflected by a number of sites during the Copper Age–for instance in present-day Bulgaria, Czech Republic and Austria–where crucibles and ingots were found far away from the copper ore sources.

### Ornament hoards

The ornament types of the Magyaregres hoard dating to the turn of the 5^th^–4^th^ millennium BCE correspond well with the items associated with the Central European metallurgical tradition [12,15,31,56]. The hoard found at Stollhof, in Austria is perhaps the most similar in its composition to the Magyaregres assemblage, containing (among others) two spiral bracelets, six spectacle spiral pendants and nine tubular spiral coils [41].

Analogous pieces to the large spiral bracelets and spectacle spiral pendants have only been documented as components of hoards or as stray finds, whose distribution concentrates in the geographical area of present-day Hungary, Slovakia, Austria and the Czech Republic [44,104,115,116]. There is very little contextual information available on the finding circumstances of these assemblages, most of them are referred to as stray finds or were discovered accidently during the 19^th^ or the beginning of the 20^th^ century (e.g., Stollhof, Štramberk-Kotouč, Malé Leváre, Rašovice) [36,44–46,89,104,117]. Apart from the Magyaregres piece, a single example for the spectacle spiral pendants is known from Hungary, the vicinity of Balassagyarmat, unfortunately, this ornament was also reported as a stray find [44]. Although smaller variants of spiral bracelets have been documented from Hungary, larger types are so far unknown from this region. Similarly to the Magyaregres hoard, the copper disc unearthed at the Balaton-Lasinja settlement of Zalavár-Basasziget is likely that it had also been deposited intentionally in a refuse pit under a building [30].

Hoards containing large spiral bracelets and tubular spiral coils were more characteristic in the subsequent chronological phase. Such assemblages occur in the territories of present-day Poland and Denmark (e.g., Rudki, Skarbienice, Bygholm) [24,33,118]. Although the Rudki hoard dates to the much later period of the Trichterbecher culture, to 3650 (68.2%) 3100 cal BCE, the design and execution of the spiral bracelet is very similar to the Magyaregres pieces [33,118].

Likewise, tubular spiral coils are known primarily from hoards from around the final phase of the 5^th^ millennium BCE, although from the 4^th^ millennium BCE they also occur in burials in the region of Central and Northern Europe. In cases where the context of wear could be identified, the shorter tubular spiral coils were usually strung as beads. The utilisation of the longer variants as dress ornaments remains unclear [24,33,41,117,119].

A hoard being deposited in a cooking pot certainly represents a rare occurrence in the Copper Age, but not entirely without example (e.g., Vanovice, Hlinsko, Skarbienice) [31,47,120]. Analogous pieces to the Magyaregres stone beads came to light–also from a cooking pot–from the site of Hornstaad-Hörnle IA, which dating overlaps with the final phase of the Magyaregres settlement [121–124]. Ornament hoards have also been documented in Cucuteni-Tripolje contexts, placed in ceramic vessels and deposited within settlements [125–128], however, the design and range of these objects are more characteristic to the Southeastern European metallurgical tradition, with assemblages clearly distinguishable from the Central European metal artefacts both geographically and typologically.

The interpretation of prehistoric hoards and the reasons why these items came to be deposited in the first place is wide ranging. Suggestions of accidentally lost items, artefacts hidden during crises, sacrifices or gifts were all considered as possible explanations. Given the context and the manner of deposition of the Magyaregres hoard, it is unlikely that they were misplaced or hurriedly hidden items, rather the situation indicates careful consideration. It has been suggested as an explanation that metal hoards represent a selection of material being returned to the ground by metal workers [129]. In the case of the Magyaregres hoard this explanation is not relevant, as the assemblage was deposited at a location where the local communities seem to have lacked the knowledge of what and how these objects were made in the first place.

However, David Fontijn’s interpretation regarding Bronze Age traditions of depositions could also be partially applicable in the Magyaregres case, as it reflects transregional selection preferences [130], in a cultural context where the use and treatment of spectacle spiral pendants and the spiral bracelets might have been strictly prescribed, and where these artefacts were probably linked to the realm of the living (i.e. the settlement) while being distanced from the realm of the dead (i.e. the burial ground). Although the communities living in the region of Transdanubia could have shared sociocultural traditions and perhaps value systems with the groups in the Northwestern Carpathians (where the ores originated and the objects were produced), there is so far no evidence for the existence of local metalworkers in southern Transdanubia. Therefore, the Magyaregres hoard could be considered as tangible evidence for interregional social networks that operated through the social interactions of a series of small communities. This leads to the question whether the hoard was linked to a single individual or an entire community, or was perhaps an assemblage complied, altered and passed down through generations.

### Burials and reconstruction of wear

The pieces of the Magyaregres hoard could undoubtedly be identified as ornaments, nevertheless, in order to reconstruct how these ornaments could have been worn or by whom proves to be difficult task. Traces of abrasion or wear linked to frequent use of the ornaments cannot be detected on either of the pieces. The burial traditions of the Jordanów/Jordansmühl-Ludanice-Balaton-Lasinja complex remain relatively obscure, compared to its extensive distribution and the number of settlements, the few known burials reflect highly diverse mortuary rituals [131]. Smaller copper and stone beads, and short tubular spiral coils are the only artefact types that appear both in burial assemblages (possibly as part of a garment or components of complex ornaments) and among the items of hoards.

Although the analysis of the Magyaregres stone beads is in progress, similar pieces are known from the contemporaneous Tiszapolgár burials on the Great Hungarian Plain, and female Ludanice burials from the area of Budapest and Slovakia, in a position implying that the beads were sewn on to belts as embellishment [58,132,133]. This type of ornament appears to be widespread across the territories of Central and Southeast Europe during the Copper Age [134,135]. Similar stone beads came to light from regions along the Danube and its tributaries in southern Germany and Switzerland, at the site of Hornstaad-Hörnle IA, where even a workshop producing stone beads was discovered [60,124,133,134].

Cylindrical beads rolled of copper sheets distributed extensively across large geographical areas in Central and Southeast Europe, and remained in use throughout a long period of time from the Late Neolithic to the Late Copper Age [109,119,125,136–141]. Necklaces, belts or head gear constructed of short cylindrical beads and tubular spiral coils occur across Central Europe, characteristically in Ludanice female and infant graves [60,109,137,142]; a tradition that continues in the subsequent chronological phase (e.g., Jordanów, Vukovár) [19].

A large spiral bracelet found *in situ* on the forearm of a Late Copper Age inhumation burial at the site of Velvary, in the Czech Republic confirms the way how these ornaments were worn in the past [143]. By this time, similar objects requiring substantial amounts of raw material are unknown from the Carpathian Basin. Instead, small-sized, spiral bracelets occur among burial assemblages of–most commonly–women in the Carpathian Basin, in contemporaneous graves with the site of Magyaregres, requiring significantly less raw material [60,133]. Smaller spiral bracelets discovered *in situ* on both wrists of an adult female at the Early Copper Age burial ground of Rákóczifalva-Bivaly-tó 1/C are similar in their appearance to the Magyaregres bracelets, however the Rákóczifalva bracelets attest for strong links with the Southeast European metallurgical complex both in their manufacture and in their raw material [144–146].

The distribution of small-sized spectacle spiral pendants and the distribution of large spectacle spiral pendants do not overlap geographically; the former distributed in regions further north and east of the distribution area of large pieces, and date to the subsequent chronological phase. Several examples are known from Poland and Germany, predominantly from female burials where small spectacle spiral pendants were placed around the head and neck [104,147]. Since large spectacle spiral pendants were not included in Copper Age burial assemblages, a stone stele discovered at the site of Sion in Switzerland could help in the reconstruction of how these pieces could have been worn. The stele depicts a spectacle spiral pendant hanging from the neck of a figure, and a dagger below its belt. Since there was no clear indication of female characteristics of biological sex, the figure is generally interpreted as a male [148]. However, the stone stele was found a long distance away from the geographical distribution of large spectacle spiral pendants, and also, they occupy different time periods. Therefore any kind of direct comparison regarding the wear of these ornaments has to be carried out with care. Nevertheless, it is reasonable to assume that large spectacle spiral pendants were associated with men, or rather a group of men within the society. Hence it can be suggested that the hoard contained elements of both male and female attires, and probably represented carefully selected or curated pieces. Regarding the types and the manufacture of copper ornaments, the elements of the hoard form a clearly distinguishable unit, without any evidence for extensive wear, indicating that the pieces were not in daily use prior deposition. However, this is not true of the stone beads among which there were examples for both intact and heavily worn pieces.

## Conclusions

The archaeological significance of the Magyaregres hoard is outstanding as this is the only copper hoard, so far, from the turn of the 5^th^–4^th^ millennium BCE in Central Europe which archaeological context is well-documented and the contents of the hoard were subjected to a wide-range of scientific analyses to reveal the provenance of the raw materials and its manufacturing technologies. Although only ten samples from the 705 copper artefacts were examined with trace elemental compositional and lead isotope analyses, the similarity of raw materials makes the results representative of the whole hoard.

The typology of the Magyaregres artefacts, the technologies involved and the provenance of raw materials all imply that these ornaments were manufactured in the territories of present-day Slovakia, in the region of the Northwest Carpathians. The geographical distribution of the stone beads corresponds with this as well, however it also points to regions further to the west.

The artefacts show remarkable similarity in their manufacture and condition, therefore it is feasible to assume that these ornaments were produced (even if not at the same time) in the same workshop, or in workshops following very similar metallurgical traditions. This uniformity perhaps indicates the cohesion of these ornaments. It is, therefore, unlikely that the pieces were compiled together by members of the community throughout a longer period of time, or were passed down through generations.

Apart from the hoard, no other copper artefact was found at the site, and there was no evidence supporting any form of metallurgical activity being carried out. In fact, there has been no evidence for metallurgical activity documented from the region of Transdanubia as a whole during the Early Copper Age, thus it is possible that the ornaments of the assemblage reached the Magyaregres site as finished products and represented exceptionally high value items. The burials associated with the settlement are still lacking–similar to many other Balaton-Lasinja sites–which make it even more difficult to link the hoard to a certain community, let alone to individuals. The large, solid copper ornaments requiring significant amounts of raw material were completely absent in the burials of the Jordanów/Jordansmühl-Ludanice-Balaton-Lasinja complex. Instead, small copper ornaments that were easier to produce and required a smaller amount of raw material–beads, rings and smaller bracelets–are found solely among grave goods.

Large spectacle spiral pendants, whose distribution can clearly be distinguished both geographically and chronologically occur in hoards exclusively, which–in the cases when the circumstances of discovery were known–were deposited in ceramic vessels at settlements or in a nearby area. This implies a set of strict sociocultural regulations of how these objects could be used and in what context. Large, spiral bracelets, along with gold, silver or copper discs with bosses are closely linked to the spectacle spiral pendants. Their occurrence in special contexts can clearly be distinguished from burials and personal possessions.

Placed in a broader, macroregional context, by the turn of the 5^th^–4^th^ millennium BCE an independent Central European metallurgical circle emerged, which, relying on the raw material resources of the Northwest Carpathians, produced typologically distinct objects by employing manufacturing techniques different from the metallurgical traditions of Southeast Europe. In other words, by this period, the technological knowledge of metallurgy was already well-established and well-developed in the region of present-day Slovakia. The details and social context of its operation are not as well-understood as of the metallurgical traditions of the Balkans due to the lack of intensive research [cf. 4].

The Magyaregres hoard, discovered in Southern Transdanubia, further away from the raw material sources, demonstrates that, for this period, the mining region of the Northwest Carpathians and the workshops that relied on it was capable of producing large quantities of objects requiring large amounts of raw materials, so that their products were accessible to communities further afield; on the other hand, the small-scale community that once lived at the site of Magyaregres had either extensive and well-maintained social networks or could offer products in exchange to access to valuable copper objects.

The Copper Age and the emergence of metallurgy have long been associated in archaeological research with the rise of elites and social inequality [e.g. 8,9], but recent years’ multidisciplinary, integrative research has increasingly questioned this connection [3,4,29]. In the settlements of small-scale farming communities in Transdanubia there are no signs of social inequality, but rather of networking and connectivity over long distances, and thus of social complexity. Exploring the details of this relationship remains a task for future research.

## Supporting information

S1 File The archaeological context of the hoard. (PDF)

S2 File Description of the artefacts. (PDF)

S3 File OxCal code for the Bayesian modelling of radiocarbon dates from Magyaregres. (PDF)
