# Supplementary material for: The provenance of the raw material and the manufacturing technology of copper artefacts from the Copper Age hoard from Magyaregres, Hungary
Source: PLoS One. 2022 Nov 23;17(11):e0278116. doi: 10.1371/journal.pone.0278116 (PMC9683617; doi:10.1371/journal.pone.0278116)
Supplement: S1 File — (PDF) [file pone.0278116.s001.pdf]

S1 File. The archaeological context of the hoard

The pit, stratigraphic unit [str.] no. 383. represented an oval, slightly elongated (along NW–SE axis) with steep sides and an almost straight base (length: 504 cm, width: 410 cm, depth: 62 cm). It was in superposition with two other features; in the south, southeast, it cut slightly into pit str. no. 315., and in the southeast into pit str. no. 300. The pit itself first appeared as a greyish brown oval patch of approx. 80 cm in diameter, with a yellowish brown fill in its centre. This yellow loessy fill turned out to be a 40–50 cm thick, and petered out towards the edges of the pit. Below this a greyish brown organic-rich layer was documented, containing the hoard in the northwestern side of the pit. Underneath the hoard a fill of similar to the greyish brown top layer was found. This all suggests that the pit had already been filling in when the hoard was placed in it and filled in with the yellowish loessy fill during a short period of time. Apart from the hoard, the pit contained only a couple of other ceramic fragments [65].
