# Supplementary material for: The provenance of the raw material and the manufacturing technology of copper artefacts from the Copper Age hoard from Magyaregres, Hungary
Source: PLoS One. 2022 Nov 23;17(11):e0278116. doi: 10.1371/journal.pone.0278116 (PMC9683617; doi:10.1371/journal.pone.0278116)
Supplement: S2 File — (PDF) [file pone.0278116.s002.pdf]

S2 File. Description of the artefacts

The description of artefacts has been published in Hungarian [65].

Ceramic vessel: Biconical cooking pot of dark grey colour with a straight-cut rim and occasional orangey brown patches on its exterior. Four vertical knob handles resembling bird's beaks were applied symmetrically on the shoulder of the vessel, two of which (on the opposite side) were pierced through in a vertical direction. The clay paste was tempered with gravel. The exterior segment above the shoulders is coated by a shiny, dark grey substance. Height: 23.5 cm, rim diameter: 13.7 cm, body diameter: 24.3 cm, weight: 2.436 kg. Inv. no. Ő.2019.8.1.1.

Cylindrical copper beads: Simple cylindrical beads made of a thin, rolled-up copper sheet. The edges of the sheet usually touch at the joint, although pieces with gaps between their edges occur too. In rare cases the edges overlap. The cross section of the sheet is rectangular. Altogether 681 pieces. Length: 1 mm–10 mm, diameter: 2.5–3 mm, weight: 0.02–0.68 g. Inv. no. Ő.2019.8.1.2, Ő.2019.8.1.8, Ő.2019.8.1.33, Ő.2019.8.1.53, Ő.2019.8.1.90, Ő.2019.8.1.146, Ő.2019.8.1.184, Ő.2019.8.1.245, Ő.2019.8.1.283, Ő.2019.8.1.296, Ő.2019.8.1.300.

Stone beads: Large, white, off-white or light grey, cylindrical beads pierced through in the middle. 86 pieces of slightly curved beads with one side cut straight, the other rounded, 9 pieces of cylindrical beads with a rhomboid-shape hole (whether this was done originally or it was a result of wear, is unclear), 7 pieces of rectangular beads with straight sides and filed long edges, 8 pieces of small, rectangular beads with a slightly curved body, 13 pieces of cylindrical, curved beads with one side flatter than the other (probably due to wear), 22 pieces of curved but flattened beads, 56 pieces of round, cylindrical beads with a slightly curved body, 53 pieces of beads with a square-shaped curved body and round edges, 6 pieces of hexagonal beads. Altogether 262 pieces. Length: 13–57 mm, diameter: 8–25 mm, weight: 1.4–37 g. Inv. no. Ő.2019.8.1.3–9, Ő.2019.8.1.11–18, Ő.2019.8.1.20, Ő.2019.8.1.28–32, Ő.2019.8.1.34–46,

Ő.2019.8.1.52, Ő.2019.8.1.54–66, Ő.2019.8.1.68–89, Ő.2019.8.1.91–94, Ő.2019.8.1.96, Ő.2019.8.1.98–110, Ő.2019.8.1.112–144, Ő.2019.8.1.147–168, Ő.2019.8.1.169–178, Ő.2019.8.1.181, Ő.2019.8.1.185–207, Ő.2019.8.1.209–223, Ő.2019.8.1.225–242, Ő.2019.8.1.246–282, Ő.2019.8.1.284–295, Ő.2019.8.1.297–299.

Small, disc-shaped stone beads: Small-sized, off-white, disc-shaped stone beads with a hole in the centre. Altogether 2 pieces. Diameter: 15 mm, thickness: 3–4 mm, diameter of hole: 4 mm. Inv.no. Ő.2019.8.1.208, Ő.2019.8.1.224.

Spondylus bead: Cylindrical bead, cut slightly diagonally at its shorter ends. Length: 23 mm, diameter: 13 mm, weight: 4.7 g. Inv. no. Ő.2019.8.1.21.

Large, disc-shaped stone bead: Large-sized, white disc-shaped stone bead. Diameter: 53 mm, thickness: 7.5 mm, diameter of hole: 4 mm, weight: 23.9 g. Inv.no. Ő.2019.8.1.24.

Spiral copper bracelet: The ornament is lentoid in cross section, covered by patches of patina in places. One end of the artefact is cut off straight – at this point, it fits together with inv. no. Ő.2019.8.1.244. bracelet. The intact end is thinned out and finished in a small spiral disc turned at right angle with the main axis of the ornament. Number of coils: 5¾, height: 70 mm, diameter: 65-80 mm, diameter of spiral disc: 13 mm, coil thickness: 3–10 mm, weight: 237 g. Inv.no. Ő.2019.8.1.145.

Spiral copper bracelet: The ornament is lentoid in cross section, covered by patches of patina in places. It was thinned out towards the ends, finished in a point at both ends. Number of coils: 7¾, height: 81 mm, diameter: 63–74 mm, the max. thickness of the coil: 9 mm, weight: 291 g. Inv.no. Ő.2019.8.1.243.

Spiral copper bracelet: The ornament is lentoid in cross section, covered by patches of patina in places. One end of the artefact was cut off straight – at this point, it fits together with inv. no. Ő.2019.8.1.145. bracelet. The other end of the ornament was thinned out and cut off straight.

Number of coils: 5⅛, height: 64 mm, diameter: 73–84 mm, thickness of the coil: 4–11 mm, weight: 212 g. Inv.no. Ő.2019.8.1.244.

Spectacle spiral copper pendant: The ornament was created by coiling a long, rounded piece of copper coil, starting from either ends into a spectacle-shaped item connected by an arching loop. The body of the coil, turns into rectangular towards the middle of the spiral discs, before becoming an oblong shape. Both centres of the spirals protrude above the surface of the discs. Width: 139 mm, height: 91 mm, thickness: max. 7 mm, weight: 284 g. Inv.no. Ő.2019.8.1.182.

Spectacle spiral copper pendant: The ornament was created by coiling a long, rounded piece of copper coil, starting from either ends into a spectacle-shaped item connected by an arching loop. The body of the coil, turns into rectangular towards the middle of the spiral discs, before becoming an oblong shape. Both centres of the spirals protrude above the surface of the discs. Width: 150 mm, height: 90 mm, thickness: max. 7 mm, weight: 338 g. Inv.no. Ő.2019.8.1.183.

Tubular spiral copper coils: 16 pieces of spiral ornaments, coiled from an elongated, rectangular copper strip. Their length varies. The copper strip was either thinned out towards the ends or was cut off straight. Inside of 11 tubular spiral coils, remains of the string were found, onto which the coils were strung [65], the identification of this material is ongoing. Length: 18–90 mm, diameter: 10–16 mm, weight: 3.8–21.8 g, Inv.no. Ő.2019.8.1.10, Ő.2019.8.1.19, Ő.2019.8.1.22–23, Ő.2019.8.1.25–27, Ő.2019.8.1.47–51, Ő.2019.8.1.67, Ő.2019.8.1.95, Ő.2019.8.1.111, Ő.2019.8.1.179–180.
