# Supplementary material for: The provenance of the raw material and the manufacturing technology of copper artefacts from the Copper Age hoard from Magyaregres, Hungary
Source: PLoS One. 2022 Nov 23;17(11):e0278116. doi: 10.1371/journal.pone.0278116 (PMC9683617; doi:10.1371/journal.pone.0278116)
Supplement: S3 File — (PDF) [file pone.0278116.s003.pdf]

S3 File. OxCal code for the Bayesian modelling of radiocarbon dates from Magyaregres

```
Plot()
 {
  Sequence()
  {
   Boundary("Magyaregres Start");
   Phase("Magyaregres")
   {
    R_Date("Poz-119996", 5430, 40);
    R_Date("Poz-120016", 5340, 35);
    R_Date("Poz-119997", 5330, 35);
    R_Date("Poz-120014", 5290, 40);
    R_Date("Poz-120015", 5260, 40);
    R_Date("Poz-120013", 5050, 40);
    Span("Magyaregres");
   };
   Boundary("Magyaregres End");
  };
 };
```
